# EGF-induced nuclear localization of SHCBP1 activates β-catenin signaling and promotes cancer progression

**DOI:** 10.1038/s41388-018-0473-z

**Published:** 2018-09-03

**Authors:** Lei Liu, Yi Yang, Shihua Liu, Tianyu Tao, Junchao Cai, Jueheng Wu, Hongyu Guan, Xun Zhu, Zhenjian He, Jun Li, Erwei Song, Musheng Zeng, Mengfeng Li

**Affiliations:** 1 Key Laboratory of Tropical Disease Control (Sun Yat-Sen University), Ministry of Education, Guangzhou, Guangdong China; 2 Department of Microbiology, Zhongshan School of Medicine, Sun Yat-Sen University, Guangzhou, Guangdong China; 3 Department of Pharmacology, Zhongshan School of Medicine, Sun Yat-Sen University, Guangzhou, Guangdong China; 4 Department of Endocrinology and Diabetes Center, The First Affiliated Hospital of Sun Yat-sen University, Guangzhou, Guangdong China; 5 Guangdong Provincial Key Laboratory of Orthopedics and Traumatology, Guangzhou, Guangdong China; 6 School of Public-Health, Sun Yat-Sen University, Guangzhou, Guangdong China; 7 Department of Biochemistry, Zhongshan School of Medicine, Sun Yat-Sen University, Guangzhou, Guangdong China; 8 Guangdong Provincial Key Laboratory of Malignant Tumor Epigenetics and Gene Regulation, Medical Research Center, Sun Yat-Sen Memorial Hospital, Sun Yat-Sen University, Guangzhou, Guangdong China; 9 State Key Laboratory of Oncology in South China, Collaborative Innovation Center for Cancer Medicine, Sun Yat-sen University Cancer Center, Guangzhou, Guangdong China

**Keywords:** Lung cancer, Cell signalling

## Abstract

Aberrant activation of EGFR represents a common event in non-small cell lung carcinoma (NSCLC) and activates various downstream signaling pathways. While EGFR activation of β-catenin signaling was previously reported, the mediating mechanism remains unclear. Our current study found that EGFR activation in NSCLC cells releases SHC-binging protein 1 (SHCBP1) from SHC adaptor protein 1 (SHC1), which subsequently translocates into the nucleus and directly promotes the transactivating activity of β-catenin, consequently resulting in development of NSCLC cell stemness and malignant progression. Furthermore, SHCBP1 promotes β-catenin activity through enhancing the CBP/β-catenin interaction, and most interestingly, a candidate drug that blocks the CBP/β-catenin binding effectively abrogates the aforementioned biological effects of SHCBP1. Clinically, SHCBP1 level in NSCLC tumors was found to inversely correlate with patient survival. Together, our study establishes a novel convergence between EGFR and β-catenin pathways and highlights a potential significance of SHCBP1 as a prognostic biomarker and a therapeutic target.

## Introduction

Lung cancer is the most commonly diagnosed cancer type and a leading cause of cancer death globally. Non-small cell lung cancer (NSCLC) accounts for approximately 85% of all lung cancer cases. Despite the availability of surgical therapy, radiotherapy, and chemotherapy, prognosis of NSCLC is still poor with overall five-year survival rate being as low as 15%, mainly due to development of resistance to chemo- and radiotherapy, postoperative recurrence and early metastasis [1–6]. Even though molecular targeted therapeutic drugs, e.g. EGFR tyrosine kinase inhibitors (TKIs), have shown encouraging efficacies on NSCLC patients in recent years, the vast majority of NSCLC patients who are initially sensitive to TKIs acquire TKI resistance and undergo relapse, metastasis, or other progressions ultimately [7, 8].

Cancer stem cells (CSCs) are subpopulations of malignant cells that possess the abilities to self-renew and differentiate within a tumor [9]. The biological properties of CSCs have been linked to tumor resistance to chemotherapy and radiation, post-treatment recurrence, and metastasis, and presumably, specific, effective CSC targeting strategies might suppress cancer relapse [10, 11]. Notably, while the molecular mechanism via which cancer cells acquire stemness and the acquired stemness is maintained remains to be understood, Wnt/β-catenin signaling has been evidently associated with the development of cellular stemness in both cancer and benign tissues [12, 13]. Canonically, activation of the Wnt/β-catenin pathway is initiated by binding of Wnt ligands to their transmembrane receptors, followed by sequestration of β-catenin in the cytoplasm away from the destined destruction complex so that β-catenin can enter the nucleus and activate transcription of its target genes, many of which have been found to contribute to the development of cellular stemess [14]. Of note, activation of β-catenin signaling has been well demonstrated in various cancer types, most of which is attributable to gene alterations of the key components of β-catenin signaling. Typically, in colorectal tumors, the vast majority (80–90%) of clinical cases contain frameshift or truncating mutations in *APC*, resulting in the loss of ability to binding β-catenin [15]. Mutations of *AXIN*, which also lead to disruption of the destruction complex, have been identified likewise. In addition, mutations of β-catenin phosphorylation sites and consequent abrogation of β-catenin phosphorylation have been found in melanoma, which leads to β-catenin accumulation in the nucleus and transcription activation of its target genes [16, 17]. In such a context, of great interest is the fact that while enhanced nuclear localization of β-catenin has been observed in NSCLC [18] and hyperactive Wnt/β-catenin signaling is associated with increased drug resistance and distant metastasis of NSCLC [19], the aforementioned mutations are rare in NSCLC [20]. Hence, the molecular mechanisms underlying the activation of the pro-stemness β-catenin signaling in NSCLC remain to be investigated.

Of note, activating mutations of EGFR are common in NSCLC. Previous reports have shown a positive correlation between the presence of activating EGFR mutations and activation of β-catenin signaling in NSCLC [21], and the convergences between these two pathways have been indicated at multiple subcellular levels [21–25]. Notably, EGFR signaling reportedly increases cytoplasmic accumulation of β-catenin and nuclear translocation by either promoting release of β-catenin from the cytoplasmic membrane or disrupting the β-catenin destruction complex [24–29]. In the meantime though, while one study reported that in U87 glioma cells EGF induced tyrosine phosphorylation of nuclear β-catenin and increased β-catenin transcription activity, little is known about the intranuclear mechanisms via which β-catenin activity is regulated by EGF–EGFR signaling.

In our present study, we show for the first time that SHC-binging protein 1 (SHCBP1), a unique protein specifically bound to the SHC1 SH2 domain and previously reported to disassociate from SHC adaptor protein 1 (SHC1) in response to EGF stimulation, mediates EGF-induced activation of β-catenin signaling in NSCLC cells. In response to EGF stimulation, SHCBP1 translocates to the nucleus, promotes interaction between β-catenin and CBP, activates β-catenin driven transcription, and enhances development of stem cell-like properties of NSCLC. These results indicate a novel convergence of the EGFR and β-catenin signaling pathways in the nucleus through nuclear SHCBP1. We also have identified that SHCBP1 may be indispensable for the stem cell-like phenotype driven by EGF-β-catenin signaling and is up-regulated in NSCLC and other cancers in a manner correlated with unfavorable clinical prognosis of the disease.

## Results

### SHCBP1 mediates EGF activation of β-catenin signaling in NSCLC

**Fig. 1:**
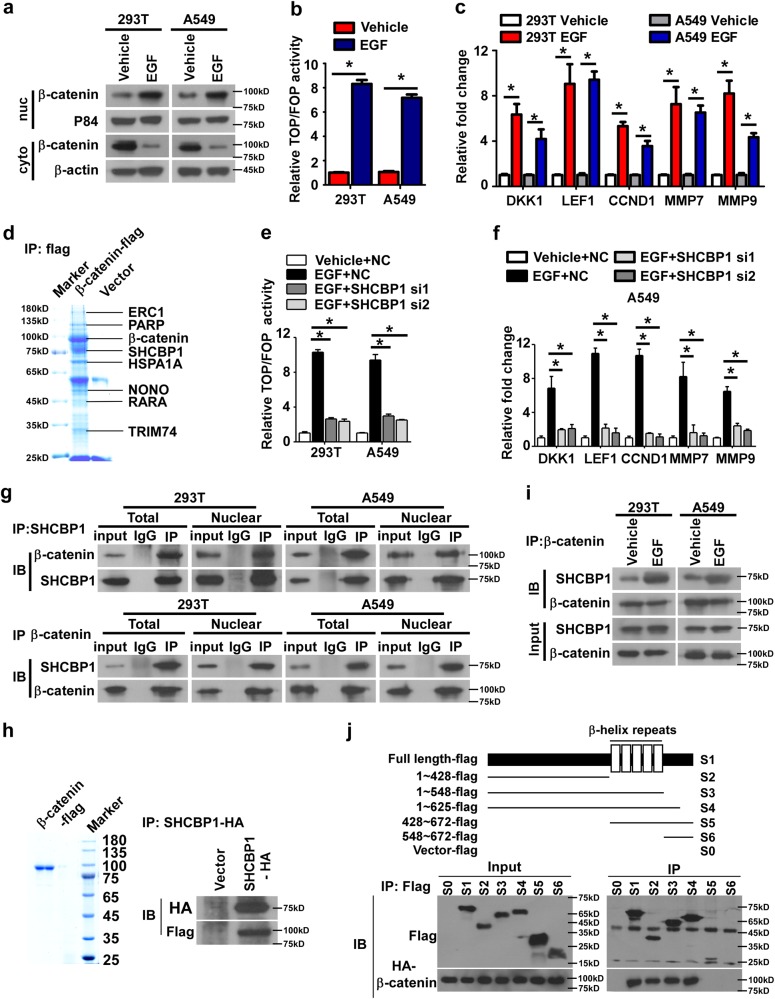
SHCBP1 mediates EGF activation of β-catenin signaling in NSCLC. **a** Western blotting assay of the nuclear and cytoplasmic extraction of 293T and A549 cells following EGF stimulation. **b** Relative luciferase activities of TOP/FOP dual-luciferase reporter determined to assess the effects of EGF (100 ng/ml) in both cell lines. **c** qRT-PCR performed to test the effects of EGF stimulation on β-catenin downstream genes in 293T and A549 cells. **d** Immunoprecipitation of FLAG- β-catenin in 293T cell lysates following EGF stimulation, and MS peptide sequencing identified SHCBP1 in the precipitate. **e** Effects of silencing SHCBP1 on cells treated with EGF, measured using TOP/FOP dual-luciferase reporter assay. **f** qRT-PCR performed to analyze the alteration of β-catenin downstream genes expression upon EGF stimulation without or with SHCBP1 depleted in A549 cells. **g** Immunoprecipitation assays to assess the interaction between β-catenin and SHCBP1 following EGF stimulation in 293T cells and A549 lung cancer cells. **h** Modified peptide pull-down assays were performed in 293T extraction using FLAG-β-catenin peptide, visualized by Coomassie staining (left panel), and incubated with SHCBP1-HA affinity agarose beads for 4 h at 4 °C. Beads containing affinity-bound proteins were washed followed by immunoblotting analysis with an anti-FLAG antibody (right panel). **i** Immunoprecipitation assays to assess the interaction between SHCBP1 and β-catenin in response to EGF stimulation. **j** Immunoprecipitation assay show that the N terminal amino-acid sequence (1–428) of SHCBP1 specifically interacts with β-catenin. For **b**, **c**, *P < 0.05 vs. Vehicle; for **e**, **f**, *P < 0.05 vs. EGF + NC

The previous finding that EGFR signaling could activate canonical Wnt/β-catenin pathways in cancer prompted us to further investigate the mechanism underlying such a crosstalk [21, 22, 25, 30]. To this goal, we began our study by examining and confirming that β-catenin could translocate to the nucleus when NSCLC cells were stimulated by EGF (Fig. 1a). Moreover, our results of TOP/FOP dual-luciferase reporter assay and expression assessment of downstream target genes verified that EGF indeed was able to activate β-catenin signaling (Fig. 1b, c) [24, 26]. Further mechanistic study was performed by immunoprecipitating (IP) FLAG-β-catenin in 293T cells extracted following EGF treatment, followed by Coomassie blue staining and mass spectrometry (MS) to identify binding partners for β-catenin. As shown in Fig. 1d, several binding partners of β-catenin have been identified, among which NONO and PARP have been found to bind β-catenin in consistence with previous reports [31, 32]. To evaluate the significance of these binding partners in EGF-activated β-catenin signaling, specific siRNA silencing of each identified binding partner was performed, and we found that only depletion of SHCBP1 markedly inhibited EGF-induced β-catenin transactivation and upregulation of the downstream genes (Fig. 1e, f and Supplementary Figs.1a–c). Notably, the interaction between SHCBP1 and β-catenin in response to EGF stimulation was verified through co-IP and western blot assays (Fig. 1g), and the specificity of such an interaction was further ascertained by modified peptide pull-down assays using FLAG-β-catenin protein purified from 293T extracts, followed by an immunoblotting assay with HA-tagged SHCBP1 antibody (Fig. 1h). Moreover, the interaction between SHCBP1 and β-catenin was markedly enhanced by EGF as evidenced by IP assay (Fig. 1i). To identify the SHCBP1 domain key to its binding with β-catenin, FLAG-tagged serially truncated SHCBP1 constructs and HA-β-catenin were co-transfected in 293T. An N terminal amino-acid sequence (1–428) in SHCBP1 was found to be pivotal for its interaction with β-catenin (Fig. 1j).

### SHCBP1 translocates to the nucleus in response to EGF stimulation

**Fig. 2:**
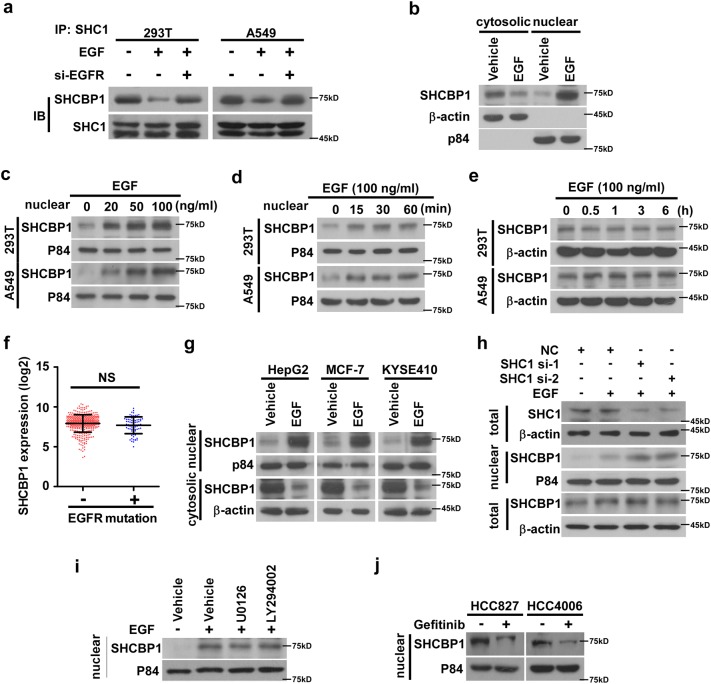
SHCBP1 translocates to the nucleus in response to EGF stimulation. **a** Following immunoprecipitation against SHC1, western blotting analysis reveals interaction between SHCBP1 and SHC1 in response to EGF treatment in the absence or presence of EGFR depletion. 293T and A549 cells were incubated with EGF (100 ng/ml) for 30 min. **b** WB analysis showing SHCBP1 redistribution in the cytoplasm and nucleus in response to EGF treatment. **c**, **d** WB examining nuclear SHCBP1 after EGF treatment at indicated concentrations (**c**) and time points (**d**) in 293T and A549 nuclear extracts. **e** Total SHCBP1 in whole-cell lysates were detected at indicated time points upon EGF treatment. **f** SHCBP1 expression in NSCLC patients with or without EGFR mutation was analyzed (data from TCGA). **g** WB results showing nuclear translocation of SHCBP1 stimulated by EGF in HepG2 liver cancer, MCF-7 breast cancer, and KYSE410 esophageal carcinoma cell lines. **h** Effects of SHC1 depletion on relocation of SHCBP1 to the nucleus. **i** WB analysis determining the impact of Ras/Erk inhibitor (U0126, 10 µM) or PI3K/Akt inhibitor (LY294002, 20 µM) on nuclear translocation of SHCBP1 in 293T cells. **j** SHCBP1 nuclear translocation was analyzed by WB assay in the absence or presence of EGFR tyrosine kinase inhibitor gefitinib in two NSCLC cell lines with EGFR activating mutations, HCC827 and HCC4006

The specific role of SHCBP1 in EGF-stimulated activation of β-catenin signaling needed to be further understood. In consistence with a previous report demonstrating that EGF triggers disassociation of SHCBP1 from adaptor protein SHC1 [33], our results showed that EGF stimulation leads to remarkable departure of SHCBP1 from SHC1 in 293T cells (Fig. 2a), and that nuclear SHCBP1 was concurrently increased (Fig. 2b–d), which could not be ascribed to expression alteration because EGF stimulation does not increase SHCBP1 expression (Fig. 2e). Of note, we also observed such an EGF-induced SHCBP1 dissociation from SHC1 and nuclear translocation without change of SHCBP1 expression in the A549 NSCLC cell lines as well (Fig. 2a, c–e), and no correlation between EGFR mutation and SHCBP1 expression could be found in The Cancer Genome Atlas (TCGA) dataset (http://cancergenome.nih.gov/) (Fig. 2f). Interestingly, the findings that nuclear translocation of SHCBP1 by EGF was also observed in cancer cell lines derived from liver, breast, and esophageal carcinomas (Fig. 2g), suggesting a possible generalizability of this biological event among different types of cancers. Furthermore, such an accumulation of SHCBP1 in the nucleus could be further enhanced by knocking down endogenous SHC1 (Fig. 2h), strongly supporting the notion that SHCBP1 indeed can be released from its complex with SHC1 and translocates to the nucleus. Since SHCBP1 changed neither the half-life of SHC1 (Supplementary Figs. 2a, b) nor the activities of the signaling pathways downstream to SHC1, i.e., the Ras/Erk and PI3K/Akt pathways, as evidenced by unaltered phosphorylation levels of ERK and Akt (Supplementary Fig. 2c), we postulated that SHCBP1 does not influence the stability and downstream signaling of SHC1. Together with the finding that the enhanced nuclear translocation of SHCBP1 was not impeded by Ras/Erk or PI3K/Akt inhibitors (Fig. 2i and Supplementary Figs. 2d, e), our data suggest that the nuclear translocation of SHCBP1 following EGF stimulation was an independent event neither affecting the quantity and regulation of SHC1 nor being affected by the canonical downstream signaling of SHC1. Notably, in two NSCLC cell lines carrying activating mutation of EGFR, HCC827, and HCC4006, we also found that the EGF-induced nuclear translocation of SHCBP1 could be blocked by pre-incubation with the EGFR TKI gefitinib (Fig. 2j), indicating that the above described events occurred in an EGFR tyrosine kinase-dependent manner. Taken together, our current data suggest that EGF stimulation can cause SHCBP1 redistribution to the nucleus.

### Nuclear SHCBP1 facilitates the binding between β-catenin and CBP and is required for EGF-induced β-catenin transactivation

**Fig. 3:**
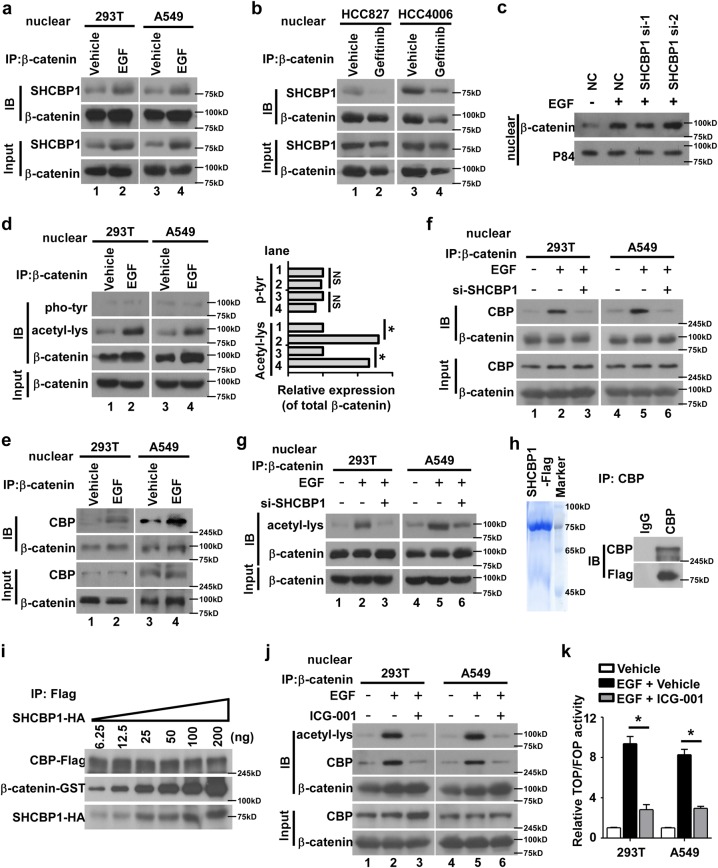
Nuclear SHCBP1 facilitates the binding between β-catenin and CBP and is required for EGF-induced β-catenin transactivation. **a** Immunoprecipitation assays showing interaction between β-catenin and SHCBP1 in the nucleus upon EGF stimulation. **b** Immunoprecipitation assays detect the effect of gefitinib on interaction between nuclear SHCBP1 and β-catenin in HCC827 and HCC4006 cells. **c** 293T cells were transfected with SHCBP1 siRNA, followed by EGF (100 ng/ml) treatment for 30 min. Nuclear extracts were analyzed by WB. **d** Immunoprecipitation assay detect the tyrosine phosphorylation and lysine acetylation of nuclear β-catenin in 293T and A549 cells treated with EGF (left panel). The strip gray level was analyzed subsequently (right panel). *P < 0.05. **e** Interaction between CBP and β-catenin upon EGF treatment was determined by immunoprecipitation assays. **f** Immunoprecipitation assay performed to examine the effects of SHCBP1 depletion on binding between CBP and β-catenin upon EGF treatment. **g** Immunoprecipitation assay performed to examine the effects of SHCBP1 depletion on lysine acetylation of β-catenin upon EGF treatment. **h** Modified peptide pull-down assays demonstrate interaction between SHCBP1 and CBP in 293T extraction using Flag-SHCBP1-peptide, visualized by Coomassie staining (left panel), incubated with a 293T extraction and followed by immunoblotting analysis with an anti-Flag antibody (right panel). **i** 293T cell lysates with CBP-Flag ectopic expression were immunoprecipitated by anti-Flag affinity gel. The products were incubated with purified β-catenin-GST protein (100 ng) and SHCBP1-HA protein at indicated concentrations for 2 h, washed for six times, and immunoblotted for Flag, GST, and HA. **j** Effects of ICG-001 on EGF-induced CBP/β-catenin interaction and lysine acetylation of β-catenin were analyzed using immunoprecipitation. **k** TOP/FOP dual-luciferase reporter assay was performed to analyze the effect of ICG-001 on β-catenin signaling activation driven by EGF. *P < 0.05 vs. EGF + Vehicle

To explore how SHCBP1 mediates EGF activation of β-catenin signaling, the effects of EGFR activity on SHCBP1/β-catenin binding were examined, and we found that EGF stimulation of EGFR in 293T or A549 cells increased SHCBP1-binding β-catenin in the nuclear extraction, while TKI treatment in HCC827 or HCC4006 NSCLC cells revealed the opposite effects (Fig. 3a, b and Supplementary Fig. 3a, b), indicating that EGFR activation indeed promotes an interaction between SHCBP1 and β-catenin. Based on this notion, we further asked whether SHCBP1 regulates β-catenin transactivation by mediating nuclear β-catenin accumulation or alternatively, post-translational modification of β-catenin, as transactivation output of β-catenin can be ascribed to these two mechanisms [34]. Our results showed that SHCBP1 depletion did not influence the distribution of β-catenin in the nucleus (Fig. 3c), suggesting that SHCBP1 is likely to regulate β-catenin activity by post-translational modifications. Interestingly, of such post-translational modifications, tyrosine phosphorylation and lysine acetylation have been found to contribute to the modulation of β-catenin signaling, as supported by a previously reported study using the U87 glioma cell line showing EGF induced tyrosine phosphorylation of nuclear β-catenin [34, 35], while our data found no change in the tyrosine phosphorylation of β-catenin in nuclear extracts of 293T and A549 cells but a remarkable enhancement of lysine acetylation in nuclear β-catenin immunoprecipitation in response to EGF stimulation (Fig. 3d). As previous reports suggested that the histone acetyltransferases CBP contribute to the acetylation of nuclear β-catenin and its transactivation [26, 36, 37], we further asked whether CBP was involved in our observed SHCBP1 mediation of EGF-induced β-catenin acetylation and activation. As exhibited in Fig. 3e and Supplementary Fig. 3c, our data showed that EGF induced binding between CBP and β-catenin in nuclear extraction of 293T and A549 cells, while silencing SHCBP1 suppressed such a binding and attenuated lysine acetylation of β-catenin (Fig. 3f, g and Supplementary Figs. 3d, e), suggesting an important role for SHCBP1 in the CBP/β-catenin interaction. Moreover, modified in vitro peptide pull-down assays showed that SHCBP1 interacted with CBP directly (Fig. 3h), and purified SHCBP1 protein significantly increased binding between CBP and β-catenin in a dose-dependent manner (Fig. 3i), indicating that SHCBP1 may enhance the affinity between CBP and β-catenin. Furthermore, when ICG-001, which binds specifically to CBP, and blocks CBP/β-catenin interaction [38], was applied, we found that it effectively repressed EGF-enhanced CBP/β-catenin interaction, β-catenin lysine acetylation, and β-catenin transactivation (Fig. 3j, k and Supplementary Figs. 3f, g). These data together suggested that the binding between CBP and β-catenin is important to EGF-stimulated activation of the β-catenin pathway.

### SHCBP1 mediates EGF-induced stem cell-like properties of NSCLC cells in vitro

**Fig. 4:**
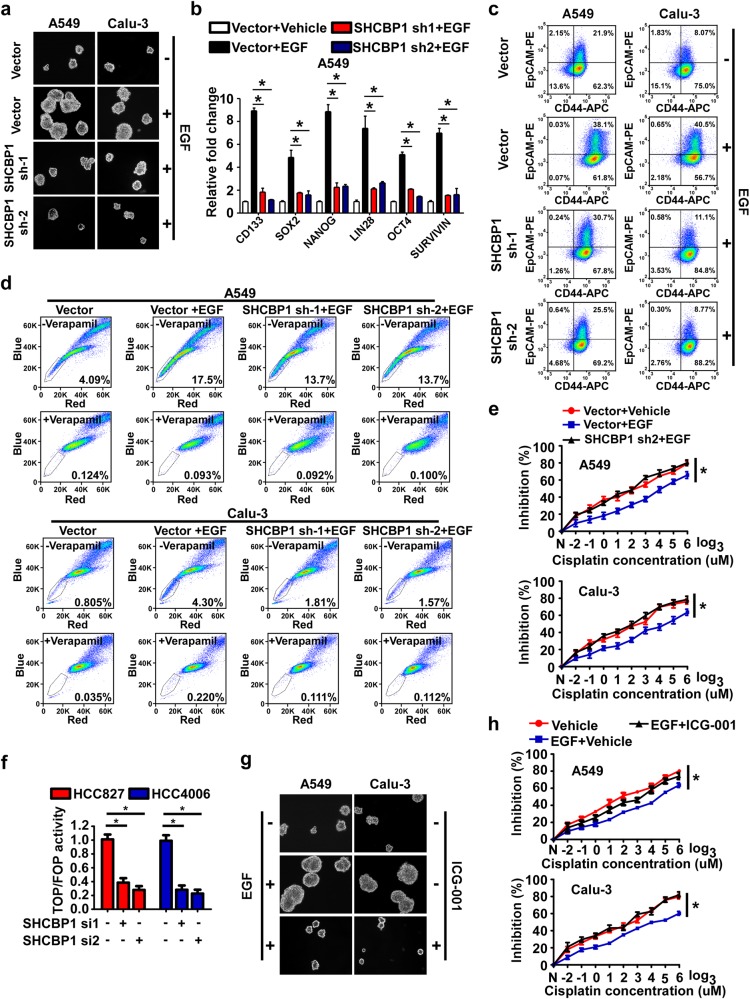
SHCBP1 mediates EGF-induced stem cell-like properties of NSCLC cells in vitro. **a** Representative images of tumor cell spheres formed by cultured cells with or without SHCBP1 silenced in the presence or absence of EGF. **b** qRT-PCR performed to assess the effects of SHCBP1 RNA interference on EGF-enhanced expression of stemness-related genes. *P < 0.05. **c** Flow cytometry determines the fraction of CD44/EpCAM double-positive cells upon EGF stimulation with or without SHCBP1 deletion. **d** Flow cytometry determines the effects of SHCBP1 depletion on SP fractions in cultured NSCLC cells treated with EGF for 48 h. **e** IC_50_ assay was carried out to assess the drug cytotoxic of cisplatin on NSCLC cell lines when SHCBP1 was silenced in the presence of EGF. *P < 0.05 vs. EGF + Vector. **f** Dual-luciferase reporter assay to assess the effects of SHCBP1 deletion on the TOP/FOP activity in HCC827 and HCC4006 cells. **g** Effects of ICG-001 on EGF-mediated increase of tumor sphere formation were analyzed. **h** IC_50_ assay was carried out to assess the cytotoxicity of cisplatin when ICG-001 was used in the presence of EGF. *P < 0.05 vs. EGF + Vehicle

The previously well-established understanding that EGF-stimulated EGFR signaling can induce and/or sustain cellular stemness under both physiological and cancer settings [39, 40], together with aforementioned role of SHCBP1 in EGF-mediated β-catenin activation as evidenced in our current study we were prompted to examine whether SHCBP1 can be involved in regulating the EGF-triggered generation of CSCs in NSCLC. As shown in Fig. 4a–d and Supplementary Figs. 4a, b, we found that depletion of SHCBP1 remarkably repressed the cellular stemness enhancement caused by EGF stimulation, as revealed by reduced EGF-promoted formation of tumor spheres, expression of stem cell markers, and CD44/EpCAM double-positive as well as SP fraction. Also notably, our data showed that silencing SHCBP1 significantly reversed the EGF-mediated upregulation of Survivin, a direct CBP/β-catenin-regulated, anti-apoptotic gene known to be involved in the maintenance of CSCs properties [36, 38, 41, 42], as well as drug resistance against Cisplatin (Fig. 4b, e, Supplementary Fig. 4b and Supplementary Table S1). Meanwhile, we also observed similar results in NSCLC cells that express activating mutation-possessed EGFR, as knockdown of SHCBP1 remarkably suppressed tumor sphere formation capacity as well as activation of β-catenin signaling (Fig. 4f and Supplementary Fig. 4c).

Further, our above-mentioned finding that EGFR activation enhances the affinity between CBP and β-catenin as well as activation of the β-catenin pathway via SHCBP1 translocation prompted us to ask whether the binding between CBP and β-catenin is also important for the stem cell properties mediated by EGF. Our results demonstrated that the ICG-001 compound effectively suppressed EGF-induced formation of tumor cell spheres, expression of stemness markers as well as Survivin, drug resistance against Cisplatin (Fig. 4g, h, Supplementary Figs. 4d, e and Supplementary Table S2), suggesting that the CBP/β-catenin interaction is important for EGF induced stem cell-like phenotype in NSCLC.

**Fig. 5:**
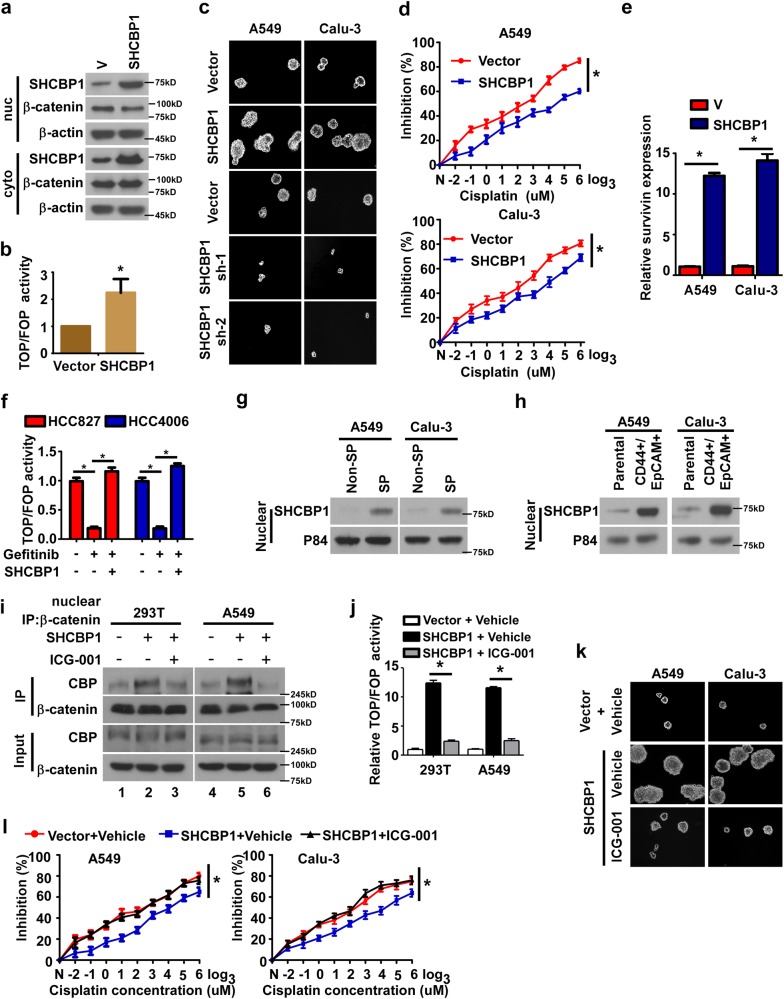
SHCBP1 upregulation per se facilitates nuclear translocation, β-catenin transactivation, and development of stem cell-like phenotype of cancer cells in vitro. **a** WB analysis showing SHCBP1 and β-catenin redistribution in the cytoplasm and the nucleus in response to ectopic SHCBP1 expression. **b** Relative luciferase activities of TOP/FOP dual-luciferase reporter assays to determine to the effects of SHCBP1 ectopic expression. *P < 0.05 vs. Vector. **c** Representative images of cultured tumor spheres in cells with SHCBP1 depletion or overexpression. **d** IC_50_ assays determine the effects of SHCBP1 overexpression on cisplatin cytotoxicity in NSCLC cells. *P < 0.05 vs. Vector. **e** qRT-PCR quantifying Survivin expression in cells with SHCBP1 ectopically expressed. *P < 0.05. **f** TOP/FOP dual-luciferase reporter assay was performed to analyze the effect of ectopic SHCBP1 expression on β-catenin signaling activity in cells treated with gefitinib. *P *<* 0.05. **g** Level of nuclear SHCBP1 was determined in NSCLC spheres and non-sphere cells by WB. **h** CD44^+^/EpCAM^+^ cells sorted from both cultured NSCLC cell lines by flow cytometry and the expression of nuclear SHCBP1 was determined by WB compared to the corresponding parental cell lines. **i** Immunoprecipitation assays to determine the effects of ICG-001 on SHCBP1-mediated CBP/β-catenin interaction. **j** TOP/FOP dual-luciferase reporter assay was performed to analyze the effect of ICG-001 on activation of β-catenin signaling driven by ectopic SHCBP1 expression. *P *<* 0.05 vs. SHCBP1 + Vehicle. **k** Representative images of tumor cell spheres cultured with or without ICG-001 treatment when the SHCBP1 was over-expressed. **l** IC_50_ assay was carried out to assess cytotoxicity of cisplatin on SHCBP1 over-expressed NSCLC cell lines in the presence of ICG-001. *P* < 0.05 vs. SHCBP1 + Vehicle

Interestingly, we observed that upregulation of SHCBP1 per se led to nuclear redistribution and β-catenin activation as well (Fig. 5a, b), and further experiments were performed to examine the significance of SHCBP1 upregulation in the development of cancer cell stemness. As shown in Fig. 5c–e, Supplementary Figs. 5a, b, and Supplementary Table S3, stable NSCLC cell lines expressing ectopic SHCBP1 displayed increased stem cell characteristics as evidenced by forming more and larger cellular spheres in suspension culture, elevated Survivin expression, and increased resistance to cisplatin. In contrast, NSCLC cell lines with SHCBP1 depleted displayed the opposite effects (Fig. 5c, Supplementary Fig. 5a–d, and Supplementary Table S4). In cells expressing EGFR possessing activating mutation, namely, HCC827 and HCC4006, when treated with TKI, ectopic SHCBP1 expression also increased SHCBP1 nuclear translocation, tumor sphere formation, and TOP/FOP activity, further suggesting that SHCBP1 overexpression-induced nuclear SHCBP1 elevation could also mediate activation of β-catenin signaling activation and increase the malignant properties of tumor cells independent of EGFR activation (Fig. 5f, Supplementary Fig. 5e, f). Moreover, higher nuclear SHCBP1 was detected in NSCLC cells cultured in serum-free medium than in serum-containing medium, as well as in CD44/ EpCAM double-positive cells compared to the corresponding parental tumor cells (Fig. 5g, h), suggesting an important role of nuclear SHCBP1 in CSCs regulation. In addition, we also found that ectopic expression of SHCBP1 stimulates EGF-induced CBP/β-catenin affinity as well as activation of the β-catenin pathway, whereas ICG-001 treatment could reverse the effects (Fig. 5i, j). Consistently, ICG-001 treatment remarkably suppressed the stemness enhancement caused by SHCBP1 upregulation (Fig. 5k, l, Supplementary Figs. 5g, and Supplementary Table S5), suggesting that nuclear SHCBP1 regulates stemness properties in a CBP/β-catenin interaction-dependent manner.

### SHCBP1-mediated cellular stemness promotes malignant phenotype of NSCLC in vivo

**Fig. 6:**
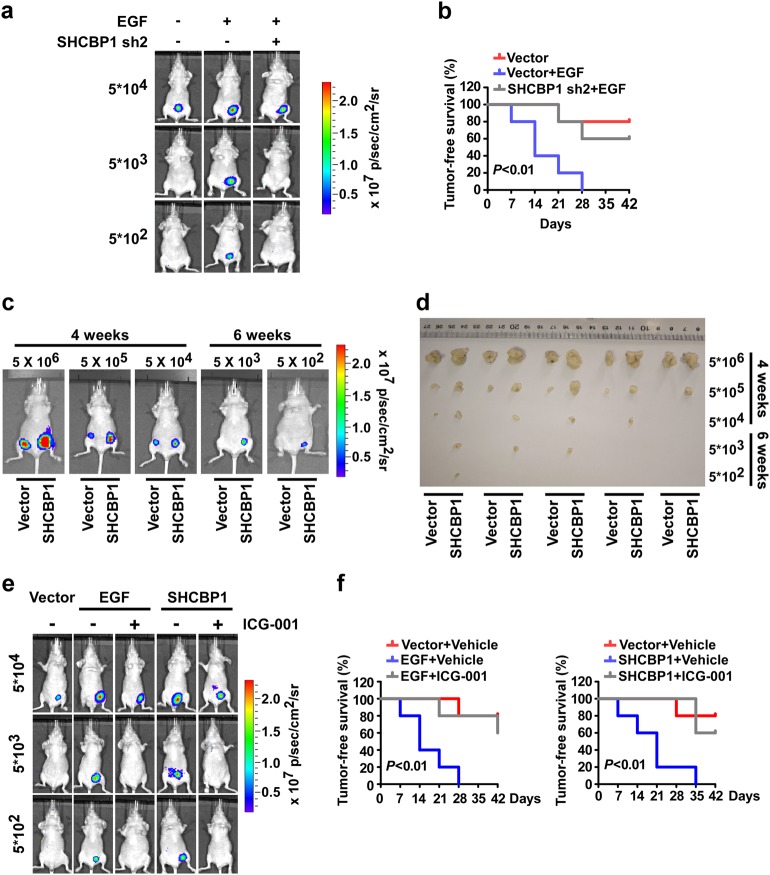
SHCBP1-mediated cellular stemness promotes malignant phenotype of NSCLC in vivo. **a** A549-luc-Vector, A549-luc-EGF, and A549-luc-EGF-sh-SHCBP1 cells at indicated numbers were implanted into the inguinal folds of nude mice. Representative bioluminescent images of subcutaneous tumors grown by indicated cells after 6 weeks are shown. **b** Tumor-free survival time was analyzed in mice with 5 × 10^4^ tumor cells implanted using in vivo bioluminescent imaging systems. **c** A549-luc-Vector and A549-luc-SHCBP1 cells at indicated numbers were implanted into the inguinal folds of nude mice. Representative bioluminescent images of subcutaneous tumors grown by indicated cells at indicated time points are shown. **d** Subcutaneous tumors at indicated time points were dissected and photographed. **e** Effects of ICG-001 on NSCLC cells with EGF or SHCBP1-ectopic expression was analyzed by subcutaneous tumorigenesis assay. ICG-001 (50 mg/kg) or vehicle (PBS) was intraperitoneally injected every 3 days for 3 weeks. **f** Tumor-free survival time was analyzed in mice with 5 × 10^4^ tumor cells implanted according to in vivo bioluminescent imaging systems

To determine whether the above described SHCBP1-mediated cellular stemness induced by EGF-EGFR signaling in vivo, we first examined the effects of ectopic SHCBP1 on the capability of human NSCLC cells to grow new tumors at low cell numbers in immunodeficient mice. We found that subcutaneous injection of as few as 5 × 10^2^ A549 cells with ectopic EGF expression led to growth of tumors, whereas at least 5 × 10^4^ control NSCLC cells were required to form a tumor (Fig. 6a). Moreover, mice injected with NSCLC cells ectopically expressing EGF had a shorter tumor-free survival time than those xenografted with control NSCLC cells (Fig. 6b). Notably, depletion of SHCBP1 markedly repressed the above alterations caused by EGF in vivo (Fig. 6a, b). Taken together, these in vivo data suggest that EGF is indeed highly capable of promoting the cellular stemness of NSCLC, and that SHCBP1 serves as an essential mediator for this EGF-promoted generation of NSCLC stem cells.

To further characterize the effect of SHCBP1 on mediating NSCLC tumorigenesis, we examined the tumorigenicity of SHCBP-overexpressing NSCLC cells at low cell numbers, and found that inoculation of as few as 5 × 10^2^ SHCBP1-A549 cells resulted in tumor growth at week 6. By contrast, at least 5 × 10^4^ vector-control A549 cells were needed to form a tumor (Fig. 6c, d, and Supplementary Table S6), altogether indicating that high-level SHCBP1 is able to promote NSCLC cell stemness and tumorigenesis (Supplementary Fig. 6).

We next asked whether the in vivo effect of SHCBP1 was associated with its role as a modulator of CBP/β-catenin signaling and thus employed the ICG-001 compound to disrupt CBP/β-catenin binding in our animal experiments. As demonstrated in Fig. 6e, f), ICG-001 effectively suppressed EGF- or high SHCBP1-induced tumorigenesis in vivo, further supporting that the CBP/β-catenin interaction is important for EGF/SHCBP1-induced stem cell-like properties in NSCLC.

### Upregulation of SHCBP1 expression in human cancers

**Fig. 7:**
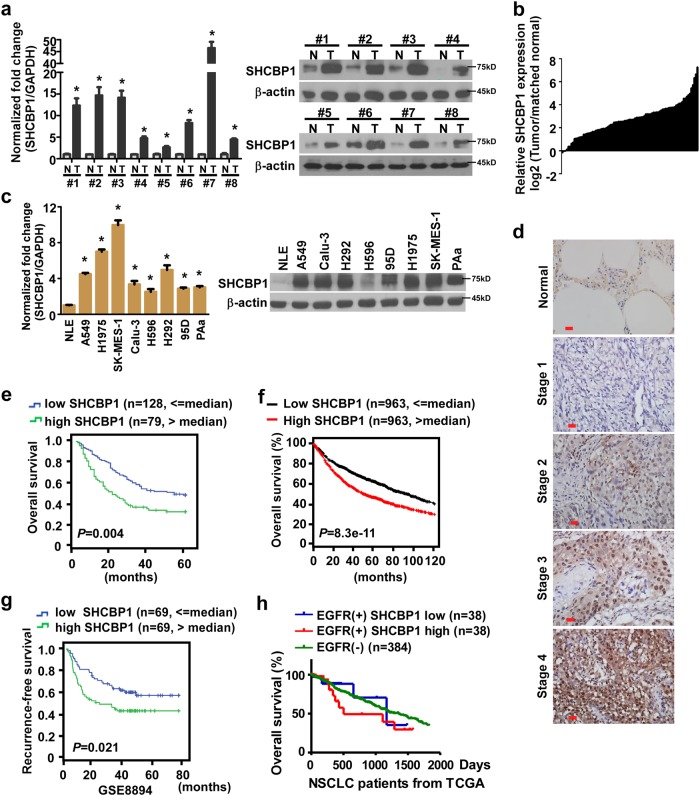
Upregulation of SHCBP1 expression in human cancers. **a** qRT-PCR and WB analyses determining the SHCBP1 mRNA (left panel) and protein (right panel) levels in eight NSCLC tumor (T) and their paired adjacent non-cancerous lung tissue specimens (N). **b** Next-generation sequencing data from The Cancer Genome Atlas (TCGA) dataset show SHCBP1 mRNA expression in 90 NSCLC tumor specimens compared to their paired adjacent non-cancerous lung tissue. **c** qRT-PCR and WB analyses to assess SHCBP1 mRNA (left panel) and protein (right panel) in indicated NSCLC cell lines as compared with benign primary lung epithelial cells (NLE). **d** Representative images of immunohistochemistry (IHC) from NSCLC patients show the protein expression level of SHCBP1 in all clinical stages of NSCLC in comparison with normal lung tissue. Scale bar: 50μm. **e** Kaplan–Meier analysis of overall survival of a cohort of 207 NSCLC patients, divided into high (>median) and low (≤median) SHCBP1 expression groups by immunohistochemistry analysis. **f** Kaplan–Meier analysis of overall survival of a cohort of 1926 NSCLC patients (data from online KMPLOT). **g** Kaplan–Meier analysis of recurrence-free survival in patients with lung cancer (GSE8894). **h** Kaplan–Meier analysis of overall survival of a cohort of 360 NSCLC patients (data from TCGA), divided into three groups according to EGFR mutation status and SHCBP1 expression

In support of its oncogenic role in NSCLC, SHCBP1 was significantly up-regulated in the eight NSCLC specimens as compared to the corresponding adjacent non-cancerous lung tissue, in both a large cohort of NSCLC patients collected in the TCGA dataset and a panel of NSCLC cell lines compared with primary normal lung epithelial (NLE) cells (Fig. 7a–c). In addition to NSCLC, SHCBP1 was also widely up-regulated in breast cancer, glioma and hepatocellular carcinoma (HCC), as shown by our analyses of the GEO datasets (GSE4290, GSE45114, and GSE29174) (Supplementary Fig. 7a), suggesting that aberrant expression of SHCBP1 might be a common event in human cancers. Moreover, elevated SHCBP1 protein level was observed in all stages of NSCLC when compared with normal lung tissue, which displayed more prominently in the nucleus as evidenced by immunohistochemistry (IHC) in clinical specimens (Fig. 7d). To further validate the clinical significance of SHCBP1 in NSCLC, clinical information collected from 207 NSCLC patients (Supplementary Table S7) was analyzed, and as expected, we found that the SHCBP1 protein level positively correlated with NSCLC clinical staging (*P* < 0.001) and T-, N-, and M-classification (*P* < 0.001, *P* = 0.035, and *p* < 0.001, respectively) (Supplementary Table S8). Furthermore, Kaplan–Meier survival analysis showed that patients bearing lung tumors with low SHCBP1 expression survived longer (median survival time = 41.5 months) than those expressing higher levels of SHCBP1 (median survival time = 30.9 months) (Fig. 7e). Consistently, Kaplan–Meier survival analysis of a larger cohort of NSCLC patients from the TCGA dataset (Supplementary Table S9) or online KMPLOT database also revealed that high SHCBP1 correlated with poor prognosis (Fig. 7f and Supplementary Figs. 7b–d). And multivariate analysis of NSCLC patients from TCGA dataset showed that SHCBP1 might represent an independent prognostic marker for NSCLC (*p* = 0.035, hazard ratio: 1.338, 95% CI, 1.021 to 1.752, Supplementary Table S10). Further analysis using the GEO dataset (GSE8894) for recurrence-free survival (RFS) found that NSCLC patients in the high SHCBP1 expression group had shorter RFS than those in the low SHCBP1 expression group (Fig. 7g). Similarly, high SHCBP1 also indicate a shorter RFS in breast cancer (GSE30682), another common cancer of epithelial origin, and liposarcoma (GSE30929), a malignant tumor of mesodermal origin (Supplementary Figs. 7e, f). Interestingly, as shown in Fig. 7h, patients bearing lung tumors with both EGFR activating mutation(s) and SHCBP1 upregulation usually had a worse prognosis. In addition, we also observed that in patients with EGFR activating mutation(s), the survival time was longer in the low- than in the high-SHCBP1 expression group, and was similar to that in the non-EGFR activating mutation group, clinically supporting the notion that SHCBP1 played an important role in EGF-mediated NSCLC malignancy.

**Fig. 8:**
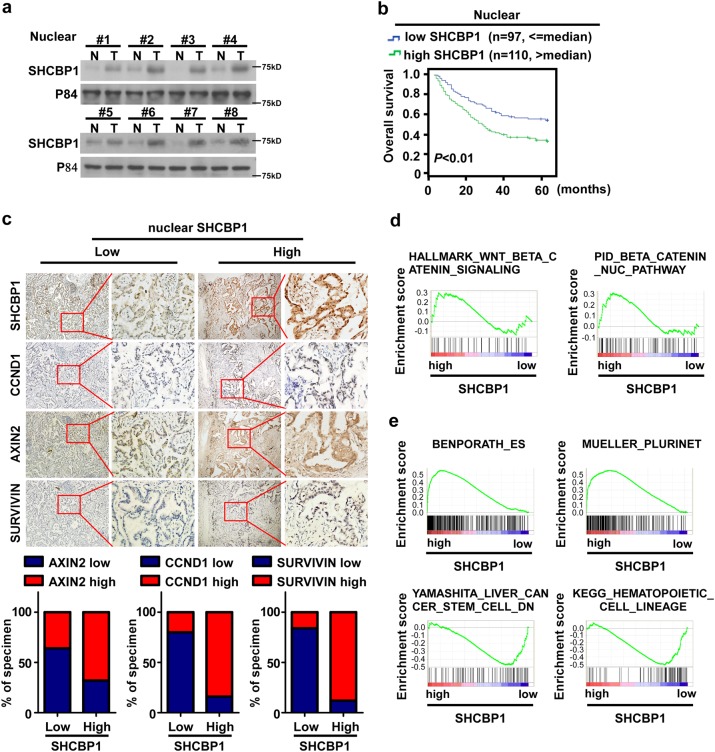
SHCBP1 expression is associated with β-catenin signaling activity and CSCs properties in NSCLC. **a** WB analyses exhibit the nuclear SHCBP1 protein levels in eight NSCLC tumor specimens (T) and their paired adjacent non-cancerous lung tissue (N). **b** Kaplan–Meier analysis of overall survival of 207 NSCLC patients which were grouped according to nuclear SHCBP1 expression assessed by immunohistochemistry analysis. **c** IHC was carried out in paraffin-embedded sections derived from 50 NSCLC patients. Correlations between nuclear SHCBP1 and Axin2, CCDN1 and survivin were analyzed by chi-squared test. **d** GSEA analysis to determine the correlation between SHCBP1 expression and β-catenin signaling activation. **e** GSEA analysis in TCGA NSCLC patients. Correlation between SHCBP1 and stemness-associated signature was identified and listed

Considering that nuclear SHCBP1 mediated EGF-induced β-catenin activation and cellular stemness, we further examined whether it was increased in NSCLC tissue. By using nuclear extraction and western blot assay, we detected far more nuclear SHCBP1 in NSCLC tumor tissues as compared with that in the corresponding adjacent non-cancerous lung tissues (Fig. 8a). Meanwhile, we found that patients bearing lung tumors with a high level of nuclear SHCBP1 had a shorter overall survival time, indicating that nuclear SHCBP1 could be a causative factor for tumor progression and represent a valuable biomarker for the prognosis of NSCLC (Fig. 8b). By performing IHC assay and chi-square test in 50 NSCLC specimens, we found that tumors with higher nuclear SHCBP1 expression usually display higher level of β-catenin signaling downstream genes Axin2, Ccnd1, and Survivin as shown in Fig. 8c, suggesting that nuclear SHCBP1 played an important role in β-catenin activation in NSCLC. Consistently, GSEA analysis of a large cohort of NSCLC patients from TCGA dataset showed that high SHCBP1 expression related to β-catenin signaling activation (Fig. 8d). In addition, we also found that SHCBP1 expression exhibited a positive correlation with stemness-related gene signature and a negative correlation with cell differentiation-related signature (Fig. 8e).

## Discussion

It is well established that in response to EGF stimulation, EGFR recruits scaffold protein SHC1 and consequently activates downstream signals, such as those mediated by Ras/Erk and PI3K/Akt pathways. This notion suggests a model in which SHC1 serves as a hub for its binding partners to trigger and/or transduce subsequent EGF–EGFR signaling and the downstream biological events. Along this context, Yong Zheng et al. [33] analyzed the timing of interaction between SHC1 and its binding proteins, and found that SHC1 first bound a group of proteins that activate cellular pro-mitogenic or survival signals and then switched to binding with proteins regulating cell invasion and morphogenesis. Interestingly, SHCBP1 had been previously found to specifically interact with the SH2 domain of SHC1 [43], and of note, while most of these SHC1 binding partners displayed enhanced affinity with SHC1 following EGF stimulation, SHCBP1 was the only molecule dissociated from SHC1 in response to EGFR activation, raising the possibility that the released SHCBP1 might play a thus far unknown role distinct from other SHC1 binding partners in the transduction and regulation of EGF–EGFR signaling [33]. In our current study, we for the first time demonstrated that upon EGF induction, SHCBP1 translocates to the nucleus where SHCBP1 binds to β-catenin and augments transactivating activity of β-catenin, leading to enhanced NSCLC cellular stemness in a SHCBP1-dependent manner. Thus, our findings have not only revealed previously unknown significance of EGF-stimulated release of SHCBP1 but more importantly also uncovered a new mode of crosstalk between the EGFR and β-catenin pathways and a novel mechanism involved in EGFR regulation of cancer cell stemness.

Notably, previous reports have shown that cytoplasmic activation of β-catenin can be regulated by EGFR signaling, leading to increased nuclear translocation of β-catenin through various extranuclear mechanisms, which either promotes release of β-catenin from the cytoplasmic membrane or accumulation of free β-catenin in the cytoplasm, or disrupts the β-catenin degradation complex so that more β-catenin enters the nucleus [21, 22, 24–29, 44–48]. Whether and how EGFR signaling could influence the DNA binding function of intranuclear β-catenin so that transcription of specific genes is activated, however, essentially remains uninvestigated. Our present study revealed that EGF-induced nuclear translocation of SHCBP1 facilitates an interaction between nuclear β-catenin and CBP, thus resulting in increased acetylation of β-catenin and subsequently activated transcription of stemness-associated genes. This finding highlighted that EGFR regulation of Wnt/β-catenin signaling may also occur in the nucleus, suggesting a multi-level feature of the crosstalk between the two, i.e., EGFR and β-catenin, pathways.

Interestingly, our data showed that disruption of CBP/β-catenin interaction with the ICG-001 compound could abrogate the EGFR enhancement of β-catenin transactivating activity. ICG-001 is an inhibitor that specifically blocks CBP/β-catenin binding. Moreover, it was found to eliminate drug-resistant tumor-initiating cells and promote differentiation of chemotherapy-insensitive CSCs by inhibiting β-catenin signaling [36, 38, 49–52]. PRI-724, as a second generation candidate drug of ICG-001, is currently under clinical evaluation for its anti-cancer efficacies against various types of solid tumors [36, 38, 52]. Our finding that ICG-001 effectively disrupts EGF- or SHCBP1-mediated CBP/β-catenin interaction and attenuates cellular stemness in NSCLC suggest that NSCLC patients with SHCBP1 upregulation might benefit from treatments of ICG-001. Thus SHCBP1 might represent a biomarking indicator for future clinical application of ICG-001 or its analog drugs.

Our data presented in this report highlight a functional significance of SHCBP1 in mediating the crosstalk between EGFR signaling and β-catenin transactivation activity. The SHCBP1 gene, located in chromosome 16q11.2, is evolutionarily conserved across species spanning from insects to mammals [53]. Previously, high SHCBP1 expression was found in embryonic development but declined during cell differentiation, and waned off in adulthood [43, 54], indicating a possibly essential role of SHCBP1 in maintaining the potential for stem cells to differentiate. Our current study revealed an aberrant upregulation of SHCBP1 in NSCLC and identified a correlation of upregulation of total as well as nuclear SHCBP1 with unfavorable outcomes for NSCLC patients, suggesting a potentially promising use of SHCBP1 as a clinically indicative biomarker for NSCLC prognosis. In support of this notion, interestingly, NSCLC patients who display both EGFR activating mutation(s) and SHCBP1 upregulation exhibit worse clinical outcome as compared with those with only EGFR activating mutation or SHCBP1 upregulation alone, suggesting a cooperative effect of EGFR activation and SHCBP1 upregulation on tumor progression. Moreover, we also found that SHCBP1 is generally up-regulated in multiple cancer types in addition to NSCLC, including glioma, HCC, and breast cancer, suggesting that upregulation of SHCBP is not an NSCLC-specific event but instead might be attributed to genetic or epigenetic mechanisms commonly active in various types of cancer. In such a context then, it is of noted that while the increased presence of SHCBP1 in the nucleus can be achieved by EGFR activation, as shown in our current data, mechanisms mediating the increase of total SHCBP1 remain unclear, warranting further studies to uncover molecules and the detailed molecular cascades via which the expression level of SHCBP1 is regulated.

In summary, this work has identified SHCBP1 as a new mediator for the crosstalk between EGFR and β-catenin signaling pathways and a new mechanism mediating the development and maintenance of NSCLC stem cells. Our data also support that SHCBP1 is a potential therapeutic target and prognostic biomarker for the disease. Future opportunity of developing anti-NSCLC strategies targeting SHCBP1/β-catenin/CBP interaction under different EGFR activity statuses is under investigation in this laboratory.

## Materials and methods

### Cell cultures

All tumor cell lines were obtained from the Cell Bank of Shanghai Institutes of Biological Sciences (Shanghai, China), Fu Erbo Biotechnology Co., Ltd (Guangzhou, China), or ATCC, and cultured as previously described [55]. Primary NLE cells were obtained and cultured in keratinocyte-SFM medium (KSFM) [55]. Authenticity of the cell lines was verified by short tandem repeat (STR) fingerprinting at the Medicine Laboratory of Forensic Medicine Department of Sun Yat-sen University (Guangzhou, China).

### Tumor specimens from patients

Clinical tissue specimens were histopathologically diagnosed at the Sun Yat-Sen University Cancer Center from 2000 to 2004. The histological characterization and clinicopathologic staging of the cases were determined by following the standard provided in the current Union for International Cancer Control (UICC) Tumor-Node-Metastasis (TNM) classification. Each tumor and adjacent non-cancerous lung tissue pair was obtained according to our previous reports [55, 56]. Prior patients’ consents and approval from the Institutional Research Ethics Committee were obtained.

### Plasmids and transfection

The SHCBP1 expression plasmid was generated by PCR subcloning the human SHCBP1 coding sequence into the lentiviral transfer plasmid pSin-puro (Clontech, Palo Alto, CA) to generate plasmid pSin-SHCBP1. To deplete SHCBP1 expression, two human shRNA sequences (sh1: GCGATTCAGAGCCTATCAA; sh2: CCATAGTGATCCATTGTCT) were cloned into the pSuper-retro-puro plasmid to generate pSuper-retro-SHCBP1-sh1 and pSuper-retro-SHCBP1-sh2. Human EGF-coding sequence was subcloned into the lentiviral transfer plasmid to generate plasmid pSin-EGF. Retroviral and lentiviral production and infection were performed according to the manufacturer’s instructions.

### RNA extraction and real-time PCR

Total RNA extraction, reverse transcription, and real-time PCR were performed as described previously [55]. Primers were purchased from Invitrogen. The cDNA was acquired by using the GoScript™ Reverse Transcription Mix (Promega). All results were normalized for the expression of GAPDH, and relative quantification was calculated using the 2^−△△CT^ formula.

### Immunoprecipitation and protein purification

Lysates were prepared from 3 × 10^7^ 293T cells transduced with Flag-tagged SHCBP1 or vector using lysis buffer. Lysates were then incubated with FLAG affinity agarose (Sigma-Aldrich, St Louis, MO) overnight at 4 °C. Beads containing affinity-bound proteins were washed six times with immunoprecipitation wash buffer. Proteins were separated on SDS polyacrylamide gels stained with Coomassie blue, and all bands were subjected to mass spectrometry analysis. SHCBP1 and β-catenin protein purification was acquired by using immunoprecipitation and FLAG/HA competing peptides (MedChemExpress, Monmouth, NJ, USA) (see Supplementary Materials and Methods for details).

### Western blotting analysis

Western blotting analysis was performed as described previously [55, 57]. The antibodies used are listed in Supplementary Information.

### Luciferase reporter assay

Cells were seeded in triplicates in 24- or 48-well plates and allowed to settle for 24 h. Indicated plasmids plus 10 ng pRL-TK renilla plasmid was transfected into the cells using the Lipofectamine 3000 reagent (Invitrogen). Forty-eight hours after transfection, Dual-Luciferase reporter assays were performed according to the manufacturer’s protocol of Dual Luciferase Reporter Assay Kit (Promega, Madison, WI). The reporter plasmids containing wild-type (CCTTTGATC; TOPflash) or mutated (CCTTTGGCC; FOPflash) TCF/LEF DNA-binding sites were purchased from Upstate Biotechnology (Lake Placid, NY).

### Nuclear and cytoplasmic extraction

NE-PER Nuclear and Cytoplasmic Extraction Reagents Kit (Thermo Scientific ™ Pierce, USA) was used and the assays were performed according to the manufacturer’s instructions.

### Primary sphere formation

Tumor spheres were cultured according to a previous report [58]. Cells were allowed to further grow for 10 days, and the numbers of spheres were microscopically counted. The sphere yields were calculated by the number of spheres derived from cells based on the initially plated 2500 cells.

### IHC assays

IHC assays in 207 NSCLC tissues were performed by using primary SHCBP1 antibody (Abgent, San Diego, CA) and quantified according to our previous report [59, 60]. The degree of immunostaining of indicated proteins was evaluated and scored by two independent observers as previously described, scoring both the proportions of positive staining tumor cells and the staining intensities. Scores representing the proportion of positively stained tumor cells was graded as: 0 (no positive tumor cells), 1 (<10%), 2 (10–50%), and 3 (>50%). The intensity of staining was determined as: 0 (no staining); 1 (weak staining = light yellow), 2 (moderate staining = yellow brown), and 3 (strong staining = brown). The staining index (SI) was calculated as the product of staining intensity × percentage of positive tumor cells, resulting in scores as 0, 1, 2, 3, 4, 6, and 9. Cutoff values for high- and low-expression of protein of interest were chosen based on a measurement of heterogeneity using the log-rank test with respect to overall survival. The optimal cutoff was identified as: the SI score of ≥4 was considered as high expression, and ≤3 as low expression.

### Animal studies

Female BALB/c-nu mice (5–6 weeks of age, 18–20 g) were purchased and housed in specific pathogen-free facilities on a 12-h light/dark cycle. All experimental procedures were approved by the Institutional Animal Care and Use Committee of Sun Yat-Sen University. At least five nude mice per group were used to ensure the adequate power and each mouse with different weight was randomly allocated. ICG-001 or vehicle has begun to be used 7 days after tumor cell injections in animals. ICG-001 (50 mg/kg) or vehicle (PBS) were intraperitoneally injected every 3 days for 3 weeks as described previously [50, 61]. For bioluminescent imaging assay, 15 min prior to imaging, mice were injected intraperitoneally (i.p.) with 150 mg/kg luciferin. Following general anesthesia, images were taken and analyzed with Spectrum Living Image 4.0 software (Caliper Life Sciences). Tumor growth was monitored weekly by in vivo imaging and photon radiance measurement, and the final monitoring was performed after 4 or 6 weeks after the injection. Bioluminescent imaging of primary tumors and metastases was performed in a blinded manner.

### Statistical analysis

Sample size was determined by power analysis to achieve a minimum effect size of 0.5 with *P* < 0.05 and all sample sizes were appropriate for assumption of normal distribution. Variance within each group of data was estimated and was similar between compared groups. Data analysis was performed by two independent investigators who were blinded to the sample groups. All statistical analyses were performed using the SPSS 13.0 (IBM) statistical software package. The Kaplan–Meier method was used to establish survival curves. The statistical significance of various variables for survival was analyzed using the Cox proportional hazards model in the multivariate analysis. Correlation between SHCBP1 expression and T-, N-, M-classification was analyzed using chi-square test. Comparisons between groups were performed with a two-tailed paired Student’s *t*-test. In all cases, *P* < 0.05 was defined as statistically significant.

## Electronic supplementary material

Supplementary figure1

Supplementary figure2

Supplementary figure3

Supplementary figure4

Supplementary figure5

Supplementary figure6

Supplementary figure7

Supplementary figure legends

Supplementary tables

Supplementary materials and methods

## References

[1] Feld R, Rubinstein LV, Weisenberger TH (1984). Sites of recurrence in resected stage I non-small-cell lung cancer: a guide for future studies. J Clin Oncol.

[2] Passlick B, Izbicki JR, Kubuschok B, Nathrath W, Thetter O, Pichlmeier U (1994). Immunohistochemical assessment of individual tumor cells in lymph nodes of patients with non-small-cell lung cancer. J Clin Oncol.

[3] Berns A (2005). Stem cells for lung cancer?. Cell.

[4] Chaffer CL, Weinberg RA (2011). A perspective on cancer cell metastasis. Science.

[5] Le Pechoux C (2011). Role of postoperative radiotherapy in resected non-small cell lung cancer: a reassessment based on new data. Oncologist.

[6] To KK, Tong WS, Fu LW (2017). Reversal of platinum drug resistance by the histone deacetylase inhibitor belinostat. Lung Cancer.

[7] Sequist LV, Joshi VA, Janne PA, Muzikansky A, Fidias P, Meyerson M (2007). Response to treatment and survival of patients with non-small cell lung cancer undergoing somatic EGFR mutation testing. Oncologist.

[8] Su KY, Chen HY, Li KC, Kuo ML, Yang JC, Chan WK (2012). Pretreatment epidermal growth factor receptor (EGFR) T790M mutation predicts shorter EGFR tyrosine kinase inhibitor response duration in patients with non-small-cell lung cancer. J Clin Oncol.

[9] Pattabiraman DR, Weinberg RA (2014). Tackling the cancer stem cells—what challenges do they pose?. Nat Rev Drug Discov.

[10] Ishizawa K, Rasheed ZA, Karisch R, Wang Q, Kowalski J, Susky E (2010). Tumor-initiating cells are rare in many human tumors. Cell Stem Cell.

[11] Reya T, Morrison SJ, Clarke MF, Weissman IL (2001). Stem cells, cancer, and cancer stem cells. Nature.

[12] Sokol SY (2011). Maintaining embryonic stem cell pluripotency with Wnt signaling. Development.

[13] Vermeulen L, De Sousa EMF, van der Heijden M, Cameron K, de Jong JH, Borovski T (2010). Wnt activity defines colon cancer stem cells and is regulated by the microenvironment. Nat Cell Biol.

[14] Clevers H (2006). Wnt/beta-catenin signaling in development and disease. Cell.

[15] Brannon AR, Vakiani E, Sylvester BE, Scott SN, McDermott G, Shah RH (2014). Comparative sequencing analysis reveals high genomic concordance between matched primary and metastatic colorectal cancer lesions. Genome Biol.

[16] Morin PJ, Sparks AB, Korinek V, Barker N, Clevers H, Vogelstein B (1997). Activation of beta-catenin-Tcf signaling in colon cancer by mutations in beta-catenin or APC. Science.

[17] Rubinfeld B, Robbins P, El-Gamil M, Albert I, Porfiri E, Polakis P (1997). Stabilization of beta-catenin by genetic defects in melanoma cell lines. Science.

[18] Akiri G, Cherian MM, Vijayakumar S, Liu G, Bafico A, Aaronson SA (2009). Wnt pathway aberrations including autocrine Wnt activation occur at high frequency in human non-small-cell lung carcinoma. Oncogene.

[19] Nguyen DX, Chiang AC, Zhang XH, Kim JY, Kris MG, Ladanyi M (2009). WNT/TCF signaling through LEF1 and HOXB9 mediates lung adenocarcinoma metastasis. Cell.

[20] Mazieres J, He B, You L, Xu Z, Jablons DM (2005). Wnt signaling in lung cancer. Cancer Lett.

[21] Suzuki M, Shigematsu H, Nakajima T, Kubo R, Motohashi S, Sekine Y (2007). Synchronous alterations of Wnt and epidermal growth factor receptor signaling pathways through aberrant methylation and mutation in non small cell lung cancer. Clin Cancer Res.

[22] Faivre EJ, Lange CA (2007). Progesterone receptors upregulate Wnt-1 to induce epidermal growth factor receptor transactivation and c-Src-dependent sustained activation of Erk1/2 mitogen-activated protein kinase in breast cancer cells. Mol Cell Biol.

[23] Tan X, Apte U, Micsenyi A, Kotsagrelos E, Luo JH, Ranganathan S (2005). Epidermal growth factor receptor: a novel target of the Wnt/beta-catenin pathway in liver. Gastroenterology.

[24] Ji H, Wang J, Nika H, Hawke D, Keezer S, Ge Q (2009). EGF-induced ERK activation promotes CK2-mediated disassociation of alpha-Catenin from beta-Catenin and transactivation of beta-Catenin. Mol Cell.

[25] Lu Z, Ghosh S, Wang Z, Hunter T (2003). Downregulation of caveolin-1 function by EGF leads to the loss of E-cadherin, increased transcriptional activity of beta-catenin, and enhanced tumor cell invasion. Cancer Cell.

[26] Roth JF, Shikama N, Henzen C, Desbaillets I, Lutz W, Marino S (2003). Differential role of p300 and CBP acetyltransferase during myogenesis: p300 acts upstream of MyoD and Myf5. EMBO J.

[27] Ma L, Zhang G, Miao XB, Deng XB, Wu Y, Liu Y (2013). Cancer stem-like cell properties are regulated by EGFR/AKT/beta-catenin signaling and preferentially inhibited by gefitinib in nasopharyngeal carcinoma. FEBS J.

[28] Fang D, Hawke D, Zheng Y, Xia Y, Meisenhelder J, Nika H (2007). Phosphorylation of beta-catenin by AKT promotes beta-catenin transcriptional activity. J Biol Chem.

[29] Ma L, Zhang G, Miao XB, Deng XB, Wu Y, Liu Y (2013). Cancer stem-like cell properties are regulated by EGFR/AKT/beta-catenin signaling and preferentially inhibited by gefitinib in nasopharyngeal carcinoma. FEBS J.

[30] Casas-Selves M, Kim J, Zhang Z, Helfrich BA, Gao D, Porter CC (2012). Tankyrase and the canonical Wnt pathway protect lung cancer cells from EGFR inhibition. Cancer Res.

[31] Sato S, Idogawa M, Honda K, Fujii G, Kawashima H, Takekuma K (2005). Beta-catenin interacts with the FUS proto-oncogene product and regulates pre-mRNA splicing. Gastroenterology.

[32] Park M, Lim JS, Lee HJ, Na K, Lee MJ, Kang CM (2015). Distinct protein expression profiles of solid-pseudopapillary neoplasms of the pancreas. J Proteome Res.

[33] Zheng Y, Zhang C, Croucher DR, Soliman MA, St-Denis N, Pasculescu A (2013). Temporal regulation of EGF signalling networks by the scaffold protein Shc1. Nature.

[34] Valenta T, Hausmann G, Basler K (2012). The many faces and functions of beta-catenin. EMBO J.

[35] Yang W, Xia Y, Ji H, Zheng Y, Liang J, Huang W (2011). Nuclear PKM2 regulates beta-catenin transactivation upon EGFR activation.. Nature..

[36] Ma H, Nguyen C, Lee KS, Kahn M (2005). Differential roles for the coactivators CBP and p300 on TCF/beta-catenin-mediated survivin gene expression. Oncogene.

[37] Takemaru KI, Moon RT (2000). The transcriptional coactivator CBP interacts with beta-catenin to activate gene expression. J Cell Biol.

[38] Emami KH, Nguyen C, Ma H, Kim DH, Jeong KW, Eguchi M (2004). A small molecule inhibitor of beta-catenin/CREB-binding protein transcription [corrected]. Proc Natl Acad Sci USA.

[39] Doetsch F, Petreanu L, Caille I, Garcia-Verdugo JM, Alvarez-Buylla A (2002). EGF converts transit-amplifying neurogenic precursors in the adult brain into multipotent stem cells. Neuron.

[40] Lee J, Kotliarova S, Kotliarov Y, Li A, Su Q, Donin NM (2006). Tumor stem cells derived from glioblastomas cultured in bFGF and EGF more closely mirror the phenotype and genotype of primary tumors than do serum-cultured cell lines. Cancer Cell.

[41] Takahashi-Yanaga F, Kahn M (2010). Targeting Wnt signaling: can we safely eradicate cancer stem cells?. Clin Cancer Res.

[42] Rebel VI, Kung AL, Tanner EA, Yang H, Bronson RT, Livingston DM (2002). Distinct roles for CREB-binding protein and p300 in hematopoietic stem cell self-renewal. Proc Natl Acad Sci USA.

[43] Schmandt R, Liu SK, McGlade CJ (1999). Cloning and characterization of mPAL, a novel Shc SH2 domain-binding protein expressed in proliferating cells. Oncogene.

[44] Togashi Y, Hayashi H, Terashima M, de Velasco MA, Sakai K, Fujita Y (2015). Inhibition of beta-Catenin enhances the anticancer effect of irreversible EGFR-TKI in EGFR-mutated non-small-cell lung cancer with a T790M mutation. J Thorac Oncol.

[45] Hwang KE, Kwon SJ, Kim YS, Park DS, Kim BR, Yoon KH (2014). Effect of simvastatin on the resistance to EGFR tyrosine kinase inhibitors in a non-small cell lung cancer with the T790M mutation of EGFR. Exp Cell Res.

[46] Nakayama S, Sng N, Carretero J, Welner R, Hayashi Y, Yamamoto M (2014). beta-catenin contributes to lung tumor development induced by EGFR mutations. Cancer Res.

[47] Krejci P, Aklian A, Kaucka M, Sevcikova E, Prochazkova J, Masek JK (2012). Receptor tyrosine kinases activate canonical WNT/beta-catenin signaling via MAP kinase/LRP6 pathway and direct beta-catenin phosphorylation. PLoS ONE.

[48] Zhang X, Zhu J, Li Y, Lin T, Siclari VA, Chandra A (2013). Epidermal growth factor receptor (EGFR) signaling regulates epiphyseal cartilage development through beta-catenin-dependent and -independent pathways. J Biol Chem.

[49] Wend P, Fang L, Zhu Q, Schipper JH, Loddenkemper C, Kosel F (2013). Wnt/beta-catenin signalling induces MLL to create epigenetic changes in salivary gland tumours. EMBO J.

[50] Gang EJ, Hsieh YT, Pham J, Zhao Y, Nguyen C, Huantes S (2014). Small-molecule inhibition of CBP/catenin interactions eliminates drug-resistant clones in acute lymphoblastic leukemia. Oncogene.

[51] He K, Xu T, Xu Y, Ring A, Kahn M, Goldkorn A (2014). Cancer cells acquire a drug resistant, highly tumorigenic, cancer stem-like phenotype through modulation of the PI3K/Akt/beta-catenin/CBP pathway. Int J Cancer.

[52] Takebe N, Miele L, Harris PJ, Jeong W, Bando H, Kahn M (2015). Targeting Notch, Hedgehog, and Wnt pathways in cancer stem cells: clinical update. Nat Rev Clin Oncol.

[53] Montembault E, Zhang W, Przewloka MR, Archambault V, Sevin EW, Laue ED (2010). Nessun Dorma, a novel centralspindlin partner, is required for cytokinesis in Drosophila spermatocytes.. J Cell Biol..

[54] Chen J, Lai F, Niswander L (2012). The ubiquitin ligase mLin41 temporally promotes neural progenitor cell maintenance through FGF signaling.. Genes Dev..

[55] Yang Y, Liu L, Zhang Y, Guan H, Wu J, Zhu X (2014). MiR-503 targets PI3K p85 and IKK-beta and suppresses progression of non-small cell lung cancer. Int J Cancer.

[56] Cai J, Wu J, Zhang H, Fang L, Huang Y, Yang Y (2013). miR-186 downregulation correlates with poor survival in lung adenocarcinoma, where it interferes with cell-cycle regulation.. Cancer Res..

[57] Su S, Liao J, Liu J, Huang D, He C, Chen F (2017). Blocking the recruitment of naive CD4(+) T cells reverses immunosuppression in breast cancer. Cell Res.

[58] Soeda A, Inagaki A, Oka N, Ikegame Y, Aoki H, Yoshimura S (2008). Epidermal growth factor plays a crucial role in mitogenic regulation of human brain tumor stem cells. J Biol Chem.

[59] Yang Y, Liu L, Cai J, Wu J, Guan H, Zhu X et al. DEPDC1B enhances migration and invasion of non-small cell lung cancer cells via activating Wnt/beta-catenin signaling. Biochem Biophys Res Commun. 450: 899-905. 10.1016/j.bbrc.2014.06.076 24971537

[60] Lian C, Wu Z, Gao B, Peng Y, Liang A, Xu C (2016). Melatonin reversed tumor necrosis factor-alpha-inhibited osteogenesis of human mesenchymal stem cells by stabilizing SMAD1 protein. J Pineal Res.

[61] Arensman MD, Telesca D, Lay AR, Kershaw KM, Wu N, Donahue TR (2014). The CREB-binding protein inhibitor ICG-001 suppresses pancreatic cancer growth. Mol Cancer Ther.

