# Supplementary figures and images for: EGF-induced nuclear localization of SHCBP1 activates β-catenin signaling and promotes cancer progression
Source: Oncogene. 2018 Sep 3;38(5):747–64. doi: 10.1038/s41388-018-0473-z (PMC6355651; doi:10.1038/s41388-018-0473-z)

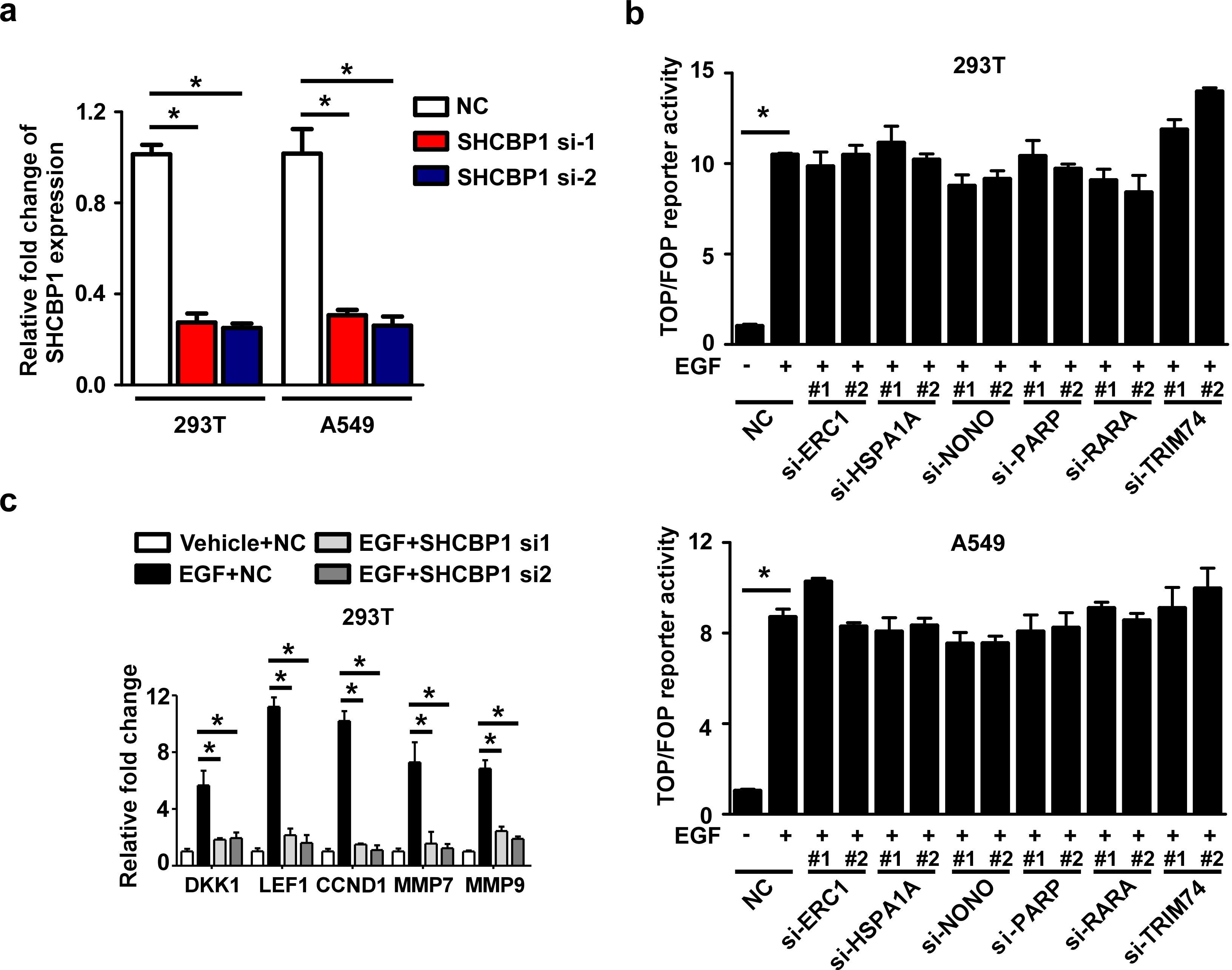

Supplement: Supplementary file 1 — Supplementary figure1 [file 41388_2018_473_MOESM1_ESM.tif]

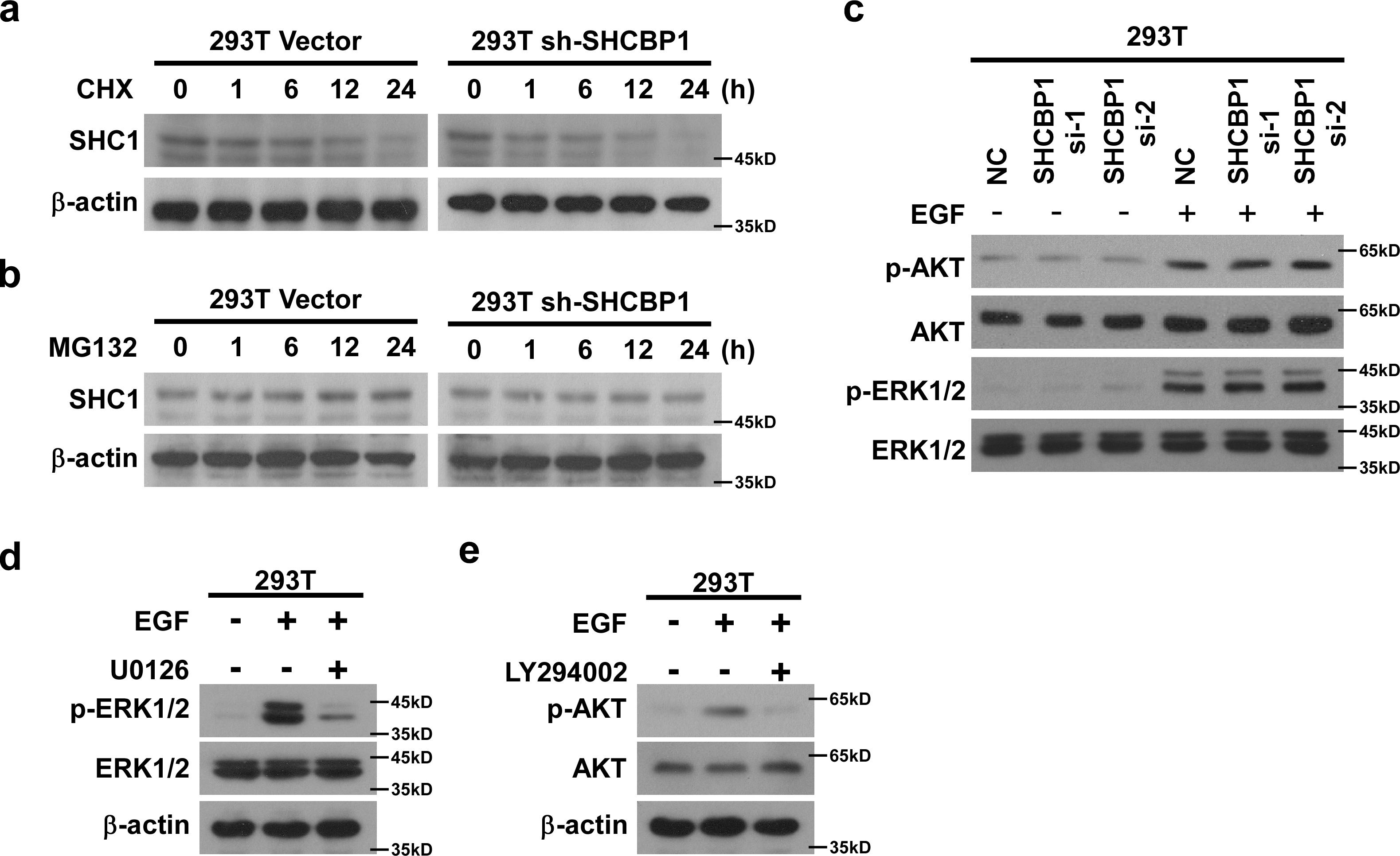

Supplement: Supplementary file 2 — Supplementary figure2 [file 41388_2018_473_MOESM2_ESM.tif]

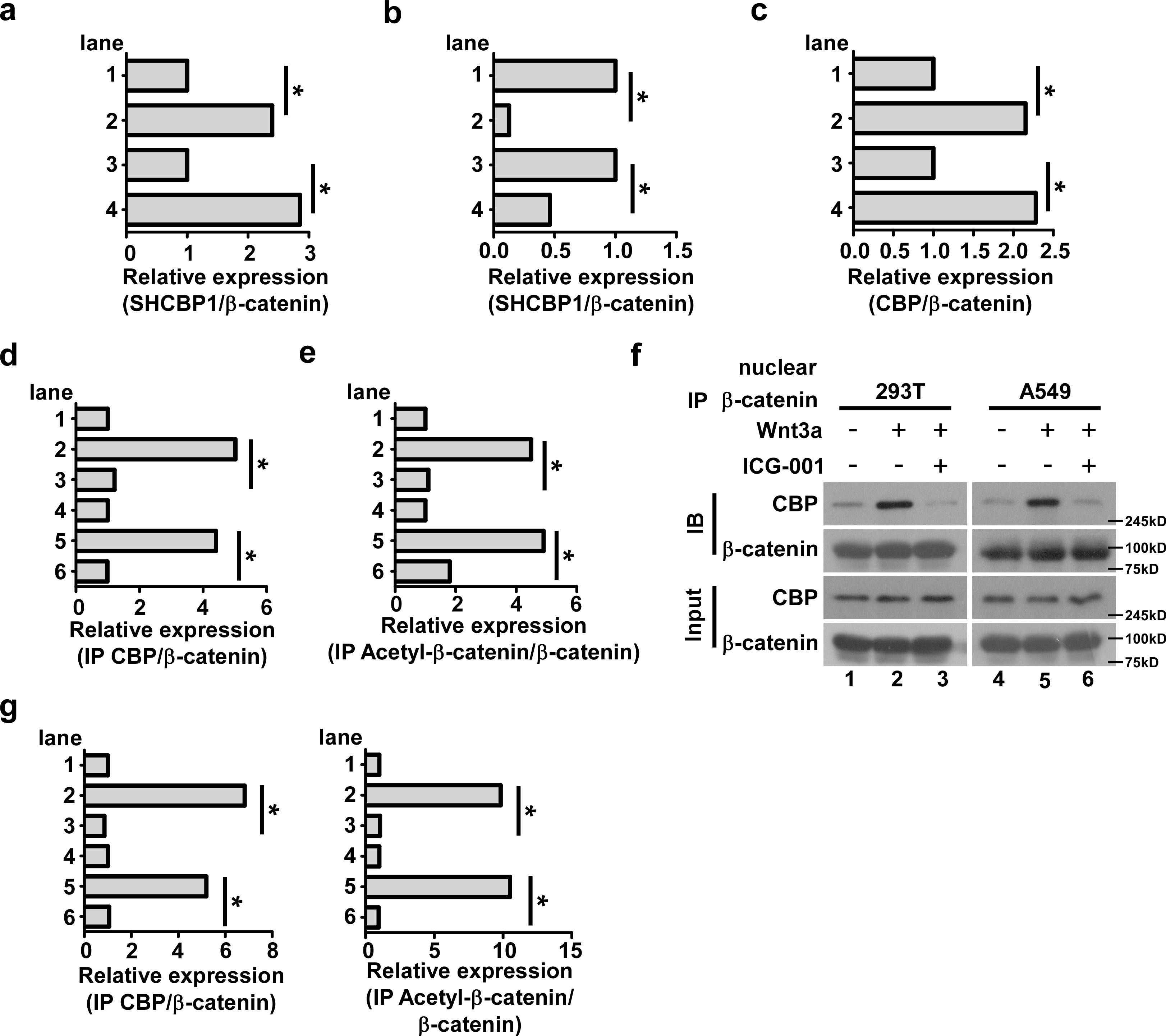

Supplement: Supplementary file 3 — Supplementary figure3 [file 41388_2018_473_MOESM3_ESM.tif]

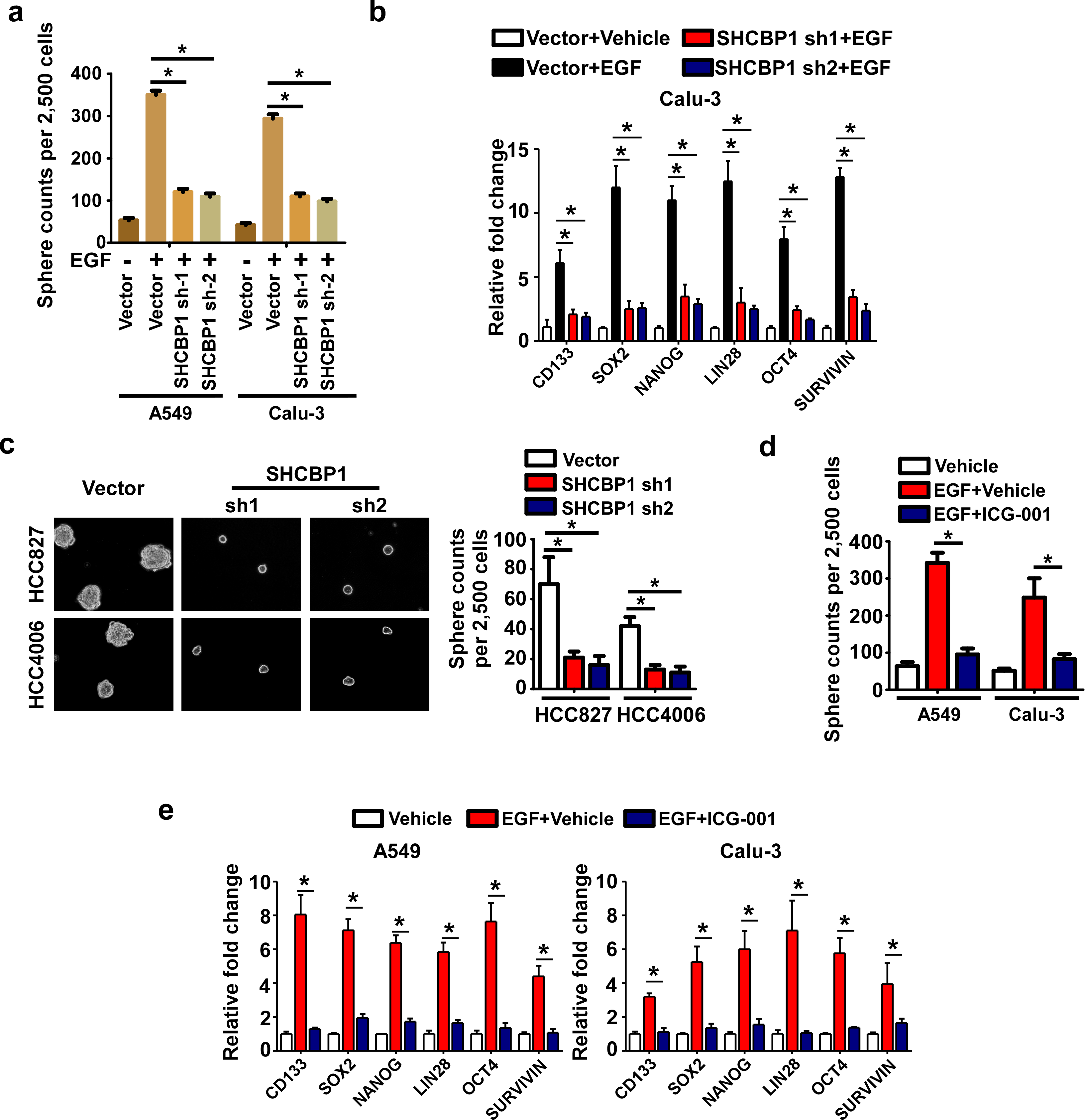

Supplement: Supplementary file 4 — Supplementary figure4 [file 41388_2018_473_MOESM4_ESM.tif]

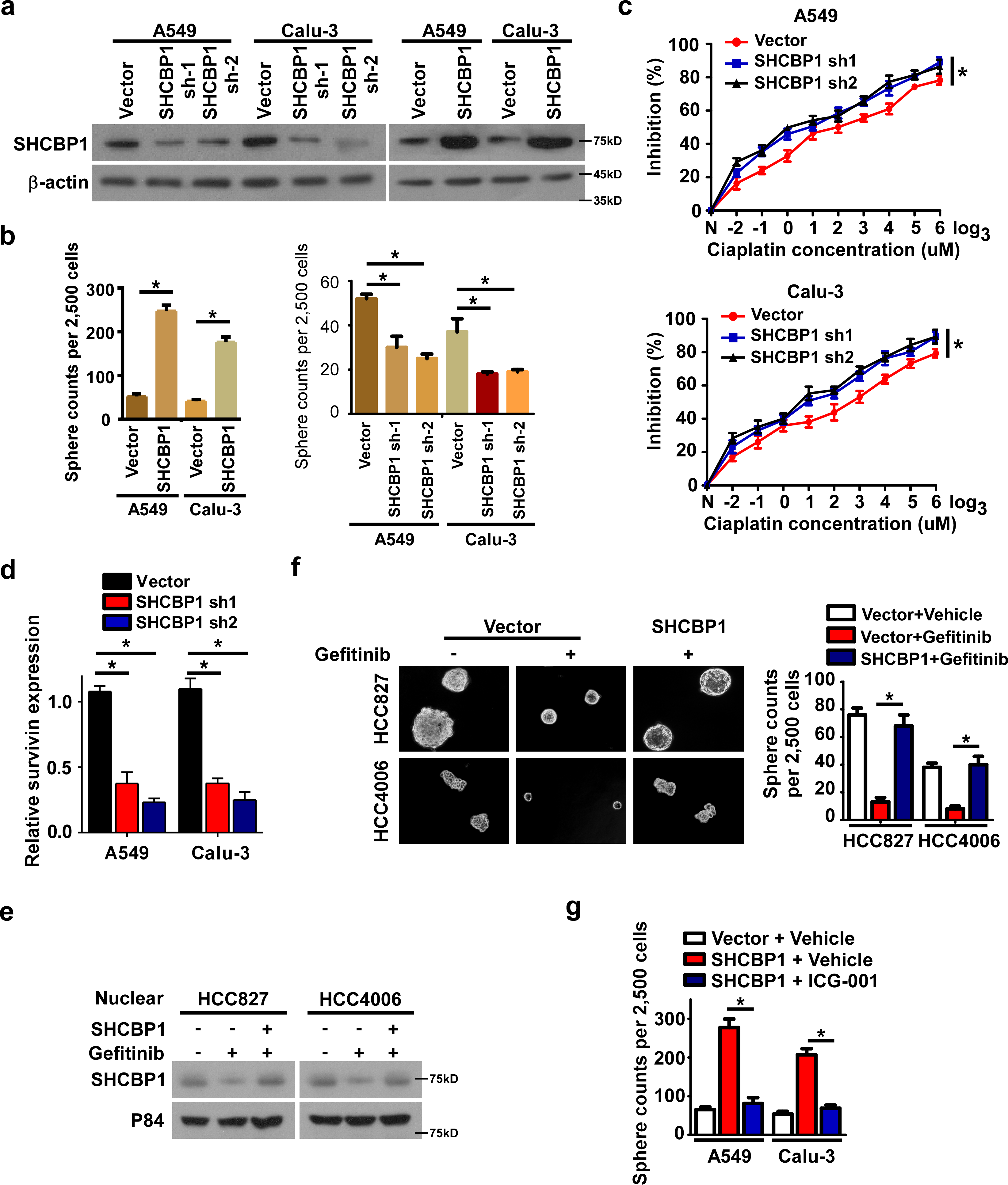

Supplement: Supplementary file 5 — Supplementary figure5 [file 41388_2018_473_MOESM5_ESM.tif]

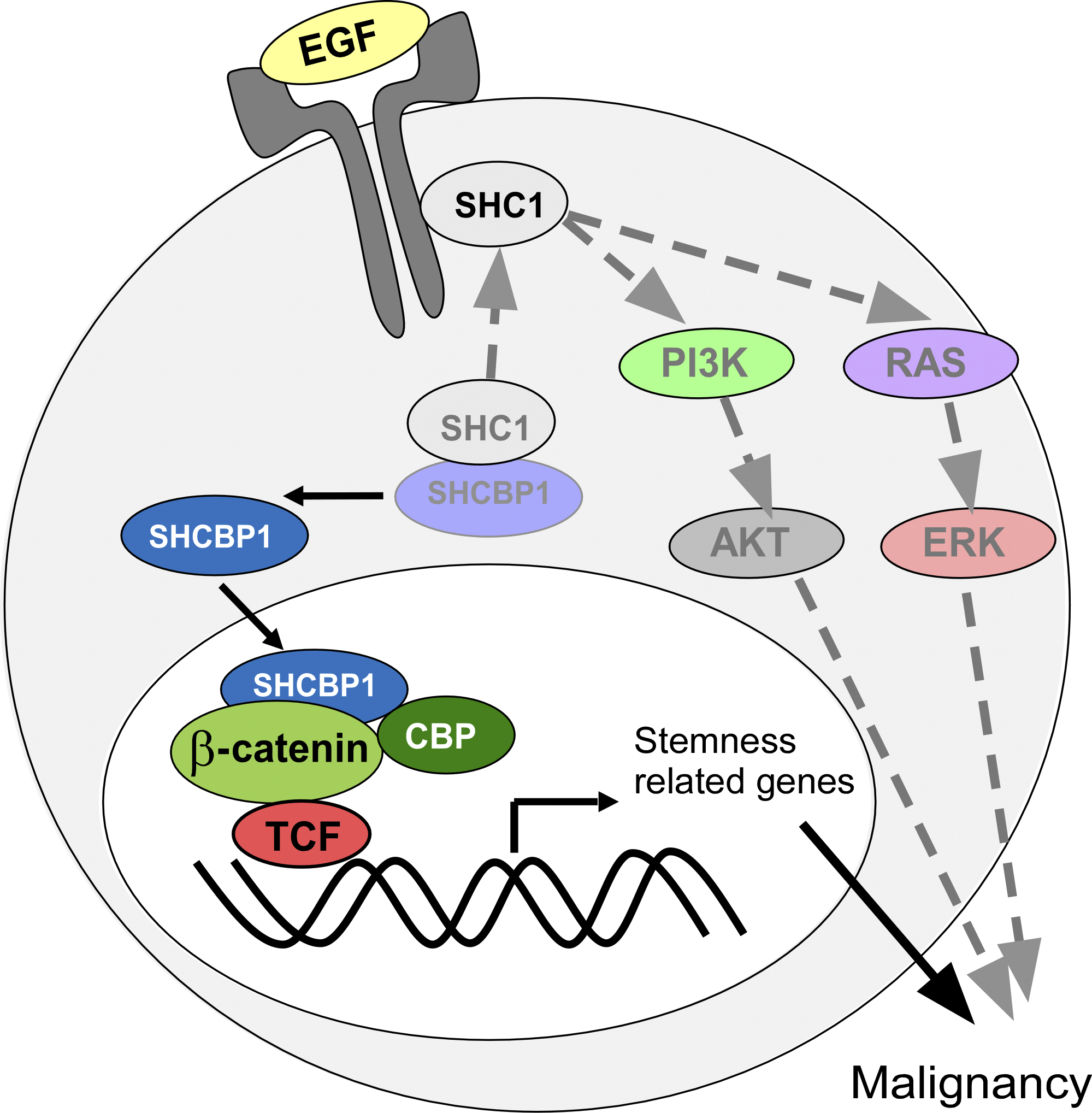

Supplement: Supplementary file 6 — Supplementary figure6 [file 41388_2018_473_MOESM6_ESM.tif]

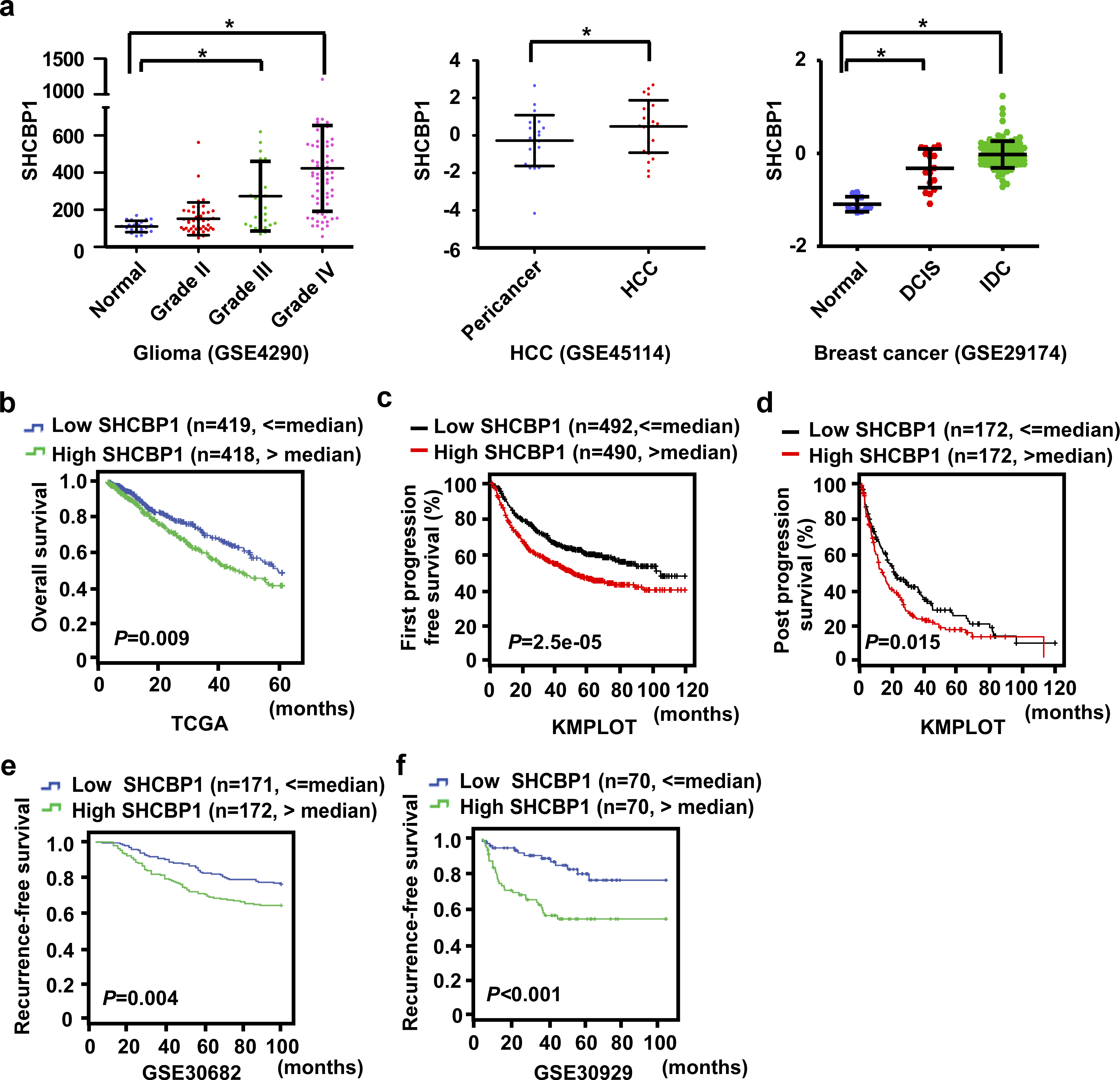

Supplement: Supplementary file 7 — Supplementary figure7 [file 41388_2018_473_MOESM7_ESM.tif]
