# Supplementary material for: EGF-induced nuclear localization of SHCBP1 activates β-catenin signaling and promotes cancer progression
Source: Oncogene. 2018 Sep 3;38(5):747–64. doi: 10.1038/s41388-018-0473-z (PMC6355651; doi:10.1038/s41388-018-0473-z)
Supplement: Supplementary file 8 — Supplementary figure legends [file 41388_2018_473_MOESM8_ESM.docx]

**Supplementary Figure Legends**

**Fig. S1. SHCBP1 mediates EGF activation of β-catenin signaling in NSCLC.** (**a**) qRT-PCR confirms the effects of SHCBP1-specific siRNAs. (**b**) TOP/FOP dual-luciferase reporter assay was performed to determine the effects of siRNAs on activation of β-catenin signaling, which specifically target β-catenin-binding partners identified in Fig 1d. (**c**) qRT-PCR performed to test the effects of EGF stimulation on β-catenin downstream genes in 293T cells. *: P < 0.05 vs. NC.

**Fig. S2 SHCBP1 translocates to the nucleus in response to EGF stimulation.**  (**a**) Stability of SHC1 protein is not affected by RNAi targeting SHCBP1 as no difference in SHC1 protein level can be detected between the SHCBP1 shRNA-transduced and NC groups in the presence of 10 μg/ml cycloheximide (CHX). (**b**) SHCBP1 interference does not change the quantity of SHC1 in the presence of proteasome degradation inhibitor, MG132 (1μM). (**c**) Phoshpo-ERK and phoshpo-Akt levels, indicating activities of Ras/Erk and PI3K/Akt, respectively, are not influenced by SHCBP1 depletion. (**d**) Efficacy of U0126 was verified by detection of phospho-ERK1/2 using WB assay in 293T cells. (**e**) Efficacy of LY294002 was verified by detection of phospho-Akt using WB assay in 293T cells.

**Fig. S3 Nuclear SHCBP1 facilitates the binding between β-catenin and CBP and is required for EGF-induced β-catenin transactivation.** (**a**) The strip gray level of Fig. 3a was analyzed. (**b**) The strip gray level of Fig. 3b was analyzed. (**c**) The strip gray level of Fig. 3e was analyzed. (**d**) The strip gray level of Fig. 3f was analyzed. (**e**) The strip gray level of Fig. 3g was analyzed. (**f**) Efficacy of ICG-001 was verified by analyzing interaction between CBP and β-catenin using immunoprecipitation. (**g**) The strip gray level of Fig. 3j was analyzed. *: P < 0.05.

**Fig. S4 SHCBP1 mediates EGF-induced stem cell-like properties of NSCLC cells *in vitro.*** (**a**) Quantification of tumor cell spheres in Fig. 4a. (**b**) qRT-PCR performed to assess the effects of SHCBP1 RNA interference on EGF-enhanced expression of stemness-related genes in Calu-3 cells. (**c**) Representative images (left) and quantification (right) of tumor cell spheres with or without SHCBP1 silenced in HCC827 and HCC4006 cells. (**d**) Quantification of tumor cell spheres in Fig. 4g. (**e**) qRT-PCR assessed the effects of ICG-001 on EGF-enhanced expression of stemness-related genes.

**Fig. S5 SHCBP1 upregulation per se facilitates nuclear translocation, β-catenin transactivation, and development of stem cell-like phenotype of cancer cells *in vitro.*** (**a**) Western blotting analysis confirms NSCLC stable cell lines with SHCBP1 ectopic expression or depletion. (**b**) Quantification of tumor cell spheres in Fig. 5c. (**c**) Effects of SHCBP1 depletion on cisplatin cytotoxicity in NSCLC cells are determined by IC50 assay. (**d**) qRT-PCR exhibiting survivin expression in cells with SHCBP1 silenced. (**e**) and (**f**) Effects of ectopic SHCBP1 expression on SHCBP1 nuclear translocation (**e**) and stemness (**f**) in HCC827 and HCC4006 cells treated with gefitinib are assessed via tumor sphere formation assay. (**g**) Quantification of tumor cell spheres in Fig. 5k.

**Fig. S6**  **Model of proposed signaling cascade.**

**Fig. S7 Upregulation of SHCBP1 expression in human cancers.** (**a**) SHCBP1 expression in glioma (GSE4290), HCC (GSE45114), and breast cancer (GSE29174). (**b**) Overall survival of NSCLC patients from TCGA. (**c**) First progression-free survival of NSCLC patients from online-KMPLOT. (**d**) Post progression survival of NSCLC patients from online-KMPLOT. (**e**) Kaplan-Meier analysis of recurrence-free survival in patients with breast cancer (GSE30682). (**f**) Kaplan-Meier analysis of recurrence-free survival in patients with liposarcoma (GSE30929).
