# Supplementary material for: EGF-induced nuclear localization of SHCBP1 activates β-catenin signaling and promotes cancer progression
Source: Oncogene. 2018 Sep 3;38(5):747–64. doi: 10.1038/s41388-018-0473-z (PMC6355651; doi:10.1038/s41388-018-0473-z)
Supplement: Supplementary file 9 — Supplementary tables [file 41388_2018_473_MOESM9_ESM.docx]

**Supplementary Tables**

**Table S1. IC50 Quantification of Fig. 4e**

|  |  | IC50 (uM) | |
| --- | --- | --- | --- |
| A549 | Vector+Vehicle | | 11.0644652 |
|  | Vector+EGF | | 97.1815308 |
|  | SHCBP1 sh2+EGF | | 8.30847243 |
| Calu-3 | Vector+Vehicle | | 11.8772226 |
|  | Vector+EGF | | 115.156363 |
|  | SHCBP1 sh2+EGF | | 9.06296237 |

**Table S2. IC50 Quantification of Fig. 4h**

|  |  | IC50 (uM) |
| --- | --- | --- |
| A549 | Vehicle | 11.08603 |
|  | EGF+Vehicle | 138.48047 |
|  | EGF+ICG-001 | 25.469248 |
| Calu-3 | Vehicle | 11.51755 |
|  | EGF+Vehicle | 118.24441 |
|  | EGF+ICG-001 | 24.963105 |

**Table S3. IC50 Quantification of Fig. 5d**

|  |  | IC50 (uM) |
| --- | --- | --- |
| A549 | Vector | 10.79511632 |
|  | SHCBP1 | 114.8140212 |
| Calu-3 | Vector | 10.67095552 |
|  | SHCBP1 | 68.29048005 |

**Table S4. IC50 Quantification of Supplementary Fig. 5c**

|  |  | IC50 (uM) |
| --- | --- | --- |
| A549 | Vector | 10.76054973 |
|  | SHCBP1 sh1 | 3.062471268 |
|  | SHCBP1 sh2 | 2.123037245 |
| Calu-3 | Vector | 11.72385935 |
|  | SHCBP1 sh1 | 3.600992004 |
|  | SHCBP1 sh2 | 2.485970443 |

**Table S5. IC50 Quantification of Fig. 5l**

|  |  | IC50 (uM) |
| --- | --- | --- |
| A549 | Vector+Vehicle | 11.259842 |
|  | SHCBP1+Vehicle | 92.7659027 |
|  | SHCBP1+ICG-001 | 13.5801251 |
| Calu-3 | Vector+Vehicle | 12.5658049 |
|  | SHCBP1+Vehicle | 95.0118101 |
|  | SHCBP1+ICG-001 | 9.81627293 |

**Table S6. Number of mice developing visible tumors when subcutaneously inoculated with indicated cells of various dosages***

| Subcutaneouse inoculation (cells) | Vector | SHCBP1 |
| --- | --- | --- |
| 5 × 10^6 a^ | 5/5 | 5/5 |
| 5 × 10^5 a^ | 4/5 | 5/5 |
| 5 × 10^4 a^ | 1/5 | 4/5 |
| 5 × 10^3 b^ | 0/5 | 3/5 |
| 5 × 10^2 b^ | 0/5 | 1/5 |

***Tumor formation was detected 4 weeks (a) and 6 weeks (b) after injection**

**Table S7. Clinicopathologic characteristics of patients enrolled for examination of SHCBP1 expression in 207 NSCLC histopathologic specimens**

|  | | **No of cases (%)** |
| --- | --- | --- |
| Sex | male | 154 (74.4) |
|  | female | 53 (25.6) |
| Age (y) | ＜60 | 118 (57.0) |
|  | ≥60 | 89 (43.0) |
| Pathologic type | Squamous cell carcinoma | 68 (32.9) |
|  | Adenocarcinoma | 86 (41.5) |
|  | Adenosquamous carcinoma | 16 (7.7) |
|  | Others | 37 (17.8) |
| Clinical staging | I | 84 (40.6) |
|  | II | 47 (22.7) |
|  | III | 59 (28.5) |
|  | IV | 17 (8.2) |
| T | T1 | 36 (17.4) |
|  | T2 | 110 (53.1) |
|  | T3 | 49 (23.7) |
|  | T4 | 12 (5.8) |
| N | N0 | 115 (55.6) |
|  | N1 | 48 (23.2) |
|  | N2 | 43 (20.8) |
|  | N3 | 1 (0.5) |
| M | M0 | 190 (91.8) |
|  | M1 | 17 (8.2) |

**Table S8. Correlation between the clinical pathologic features and expression of SHCBP1 levels**

| **Characteristics** | | **SHCBP1** | | ***P*-value** |
| --- | --- | --- | --- | --- |
|  |  | Low | High |  |
| Gender | male | 92 | 62 | 0.290 |
|  | female | 36 | 17 |  |
| Age (y) | ＜60 | 80 | 38 | 0.045 |
|  | ≥60 | 48 | 41 |  |
| Pathologic type | Squamous cell carcinoma | 42 | 26 | 0.355 |
|  | Adenocarcinoma | 48 | 38 |  |
|  | Adenosquamous carcinoma | 12 | 4 |  |
|  | Others | 26 | 11 |  |
| Clinical staging | I | 66 | 18 | <0.001 |
|  | II | 34 | 13 |  |
|  | III | 25 | 34 |  |
|  | IV | 3 | 14 |  |
| T | T1 | 26 | 10 | <0.001 |
|  | T2 | 76 | 34 |  |
|  | T3 | 24 | 25 |  |
|  | T4 | 2 | 10 |  |
| N | N0 | 79 | 36 | 0.035 |
|  | N1 | 29 | 19 |  |
|  | N2 | 19 | 24 |  |
|  | N3 | 1 | 0 |  |
| M | M0 | 125 | 65 | <0.001 |
|  | M1 | 3 | 14 |  |

**Table S9. Clinicopathologic characteristics of 837 NSCLC cases in TCGA**

|  | | **No of cases (%)** |
| --- | --- | --- |
| Sex | Male | 494 (59.0) |
|  | Female | 343 (41.0) |
| Age (y) | ≤60 | 224 (26.8) |
|  | ＞60 | 613 (73.2) |
| Pathologic type | SCC | 401 (47.9) |
|  | ADC  Low | 436 (52.1) |
| SHCBP1 | Low | 419 (50.1) |
|  | High | 418 (49.9) |
| Clinical staging ^a^ | I | 437 (52.5) |
|  | II | 217 (26.1) |
|  | III | 150 (18.0) |
|  | IV | 29 (3.5) |
| T ^b^ | T1 | 233 (27.9) |
|  | T2 | 483 (57.8) |
|  | T3 | 82 (9.8) |
|  | T4 | 37 (4.4) |
| N ^c^ | N0 | 527 (64.1) |
|  | N1 | 187 (22.7) |
|  | N2 | 101 (12.3) |
|  | N3 | 7 (0.9) |
| M ^d^ | M0 | 633 (95.8) |
|  | M1 | 28 (4.2) |

^a^: Clinical staging information of 4 patients is not provided.

^b^: T classification information of 2 patients is not provided.

^c^: N classification information of 15 patients is not provided.

^d^: M classification information of 176 patients is not provided.

**Table S10. Univariate and multivariate analyses of various prognostic variables for overall survival in 837 NSCLC cases in TCGA**

|  | Univariate analysis | |  | Multivariate analysis | |
| --- | --- | --- | --- | --- | --- |
|  | P-value | Hazard ratio (95% CI) |  | P-value | Hazard ratio (95% CI) |
| Age  ≤60  ＞60 | 0.577 | 1.091 (0.804-1.480) |  |  |  |
| Gender  Male  Female | 0.348 | 0.881 (0.675-1.149) |  |  |  |
| Pathologic type  SCC  ADC | 0.998 | 1.000 (0.694-1.443) |  |  |  |
| Stage  I  II  III  IV | ＜0.001 | 1.492 (1.304-1.707) |  | ＜0.001 | 1.468 (1.279-1.686) |
| T  T1  T2  T3  T4 | ＜0.001 | 1.455 (1.235-1.715) |  |  |  |
| N  N0  N1  N2  N3 | ＜0.001 | 1.465 (1.254-1.712) |  |  |  |
| M  M0  M1 | 0.050 | 1.668 (1.001-2.778) |  |  |  |
| SHCBP1  Low  High | 0.009 | 1.419 (1.090-1.846) |  | 0.035 | 1.338 (1.021-1.752) |
