# Supplementary material for: EGF-induced nuclear localization of SHCBP1 activates β-catenin signaling and promotes cancer progression
Source: Oncogene. 2018 Sep 3;38(5):747–64. doi: 10.1038/s41388-018-0473-z (PMC6355651; doi:10.1038/s41388-018-0473-z)
Supplement: Supplementary file 10 — Supplementary materials and methods [file 41388_2018_473_MOESM10_ESM.docx]

**Supplementary Materials and Methods**

**RNA extraction and real-time PCR**

Primers used are listed as the following:

SHCBP1-Sense: 5’-GAGTCACAGTGCGGACATCA-3’;

SHCBP1-Antisense: 5’-GCACTGACTCCCAGGGTAGA-3’;

CD133-Sense: 5’-CCATTGGCATTCTCTTTGAA-3’;

CD133-Antisense: 5’-TTTGGATTCATATGCCTTCTGT-3’;

SOX2-Sense: 5’-AACCCCAAGATGCACAACTC-3’;

SOX2-Antisense: 5’-GCTTAGCCTCGTCGATGAAC-3’;

NANOG-Sense: 5’-GATTTGTGGGCCTGAAGAAA-3’;

NANOG-Antisense: 5’-ATGGAGGAGGGAAGAGGAGA-3’;

LIN28-Sense: 5’-CCCAGTGGATGTCTTTGTGC-3’;

LIN28-Antisense: 5’-CAGACCCTTGGCTGACTTCT-3’;

OCT4-Sense: 5’-GTGGAGGAAGCTGACAACAA-3’;

OCT4-Antisense: 5’-GGTTCTCGATACTGGTTCGC-3’;

SURVIVIN-Sense: 5’- CTTTCTCCGCAGTTTCCTCA-3’;

SURVIVIN-Antisense: 5’- TTGGTGAATTTTTGAAACTGGA-3’;

DKK1-Sense: 5’-ATGCGTCACGCTATGTGCT-3’;

DKK1-Antisense: 5’-TTTCCTCAATTTCTCCTCGG-3’;

LEF1-Sense: 5’-TGGATCTCTTTCTCCACCCA-3’;

LEF1-Antisense: 5’-CACTGTAAGTGATGAGGGGG-3’;

CCND1-Sense: 5’-TCCTCTCCAAAATGCCAGAG-3’;

CCND1-Antisense: 5’-GGCGGATTGGAAATGAACTT-3’;

MMP7-Sense: 5’-GAGCTACAGTGGGAACAGGC-3’;

MMP7-Antisense: 5’-GCATCTCCTTGAGTTTGGCT-3’;

MMP9-Sense: 5’-ACGACGTCTTCCAGTACCGA-3’;

MMP9-Antisense: 5’-TTGGTCCACCTGGTTCAACT-3’

**Purification of SHCBP1 and β-catenin proteins from 293T cells**

FLAG-tagged SHCBP1 and β-catenin were expressed in a mammalian cell expression system. SHCBP1 and β-catenin recombinant proteins were purified and assessed using SDS-PAGE and Coomassie blue staining. Approximately 5×10^7^ 293T cells transfected with 200 µg pSin plasmid expressing each protein were lysed using lysis buffer (150 mM NaCl, 10 mM HEPES, 1 mM EDTA, 1 mM EGTA, 10% glycerol, pH 7.4, 1% NP-40). Lysates were then incubated with 200 μl FLAG affinity agarose (Sigma-Aldrich, St. Louis, MO) overnight at 4°C. Beads containing affinity-bound proteins were washed six times with 5 ml wash buffer (300 mM NaCl, 20 mM HEPES, 1 mM EDTA, 1 mM EGTA, 2% glycerol, pH 7.4, 0.1% NP-40), followed by elution twice with 500 μl of FLAG competing peptides (Sigma-Aldrich, 0.1μg/μl). The elutes were pooled and washed with 5 ml PBS using 3 -kDa MW cut-off filter units (Millipore) to remove the competing peptides. The purities of the recombinant protein domains were examined by SDS-PAGE and Coomassie blue staining.

**Western blotting analysis**

Antibodies used in our study include anti-SHCBP1 (Abgent, San Diego, CA), anti-β-catenin (BD Biosciences, San Jose, CA), anti-CBP (Cell Signaling, Danvers, MA), anti-SHC1 (Abcam, Cambridge, MA), anti-p84 (Abcam, Cambridge, MA), anti-p-Erk (Epitomics, Burlingame, CA), anti-p-Akt (Epitomics, Burlingame, CA), anti-p-Tyr (Cell Signaling, Danvers, MA), anti-acetyl-Lys (Cell Signaling, Danvers, MA), anti-GST (Abcam, Cambridge, MA) and anti-β-actin (Sigma-Aldrich, St. Louis, MO) antibodies. Antibodies against FLAG, GST and HA were purchased from Sigma (Sigma-Aldrich, St. Louis, MO). All Western blotting assays were independently repeated for at least three times.

**Study approval**

All animal experimental procedures were approved by the Institutional Animal Care and Use Committee of Sun Yat-sen University. The use of NSCLC donors’ samples were approved by the Institutional Research Ethics Committees of Sun Yat-sen University. Donors provided prior written informed consent.
